# High‐Efficiency Precipitate Recycling Strategy Paves the Way for Efficient Sb_2_S_3_ Solar Cells Fabricated by CBD Method

**DOI:** 10.1002/advs.76438

**Published:** 2026-07-11

**Authors:** Yeyang Lin, Zixian Cai, Mingen Zheng, Ye Huang, Weihuang Wang, Qiqiang Zhu, Qing Gao, Jianhui Chen, Shuying Cheng

**Affiliations:** ^1^ Institute of Micronano Devices & Solar Cells College of Physics & Information Engineering Fuzhou University Fuzhou China; ^2^ Jiangsu Collaborative Innovation Center for Photovoltaic Science and Engineering Changzhou People's Republic of China; ^3^ Hebei Key Lab of Optic‐Electronic Information and Materials Department of Physics Science and Technology Hebei University Baoding China

**Keywords:** chemical bath deposition, crystallization, recycling, Sb_2_S_3_, solar cells

## Abstract

Sb_2_S_3_ solar cells have emerged as a promising candidate for next‐generation photovoltaics. However, the undesirable crystallization of the absorber layer largely hindered the performance of Sb_2_S_3_ solar cells. Herein, an innovative precipitate recycling (PR) strategy is proposed to regulate the deposition of Sb_2_S_3_ thin films and the subsequent device performance. As a result, this PR strategy enabled high‐ efficiency recycling of raw materials in the traditional CBD method. More importantly, the NH_3_‐EG solution of Sb_2_S_3_ precipitates can effectively regulate the morphology of the precursor film, facilitating the crystallization of Sb_2_S_3_ film by promoting thermal diffusion and elemental migration during the post‐annealing process. Consequently, the PR strategy significantly enhanced film crystallinity and [*hk*1] orientation, optimized the CBO of CdS/Sb_2_S_3_ interface to −0.17 eV, reduced the back barrier by 35%, and defect concentration by nearly one order of magnitude (converting V_S2_ donor defect to S_Sb2_ acceptor defect). Finally, benefiting from the optimized *J*_SC_ and FF, the power conversion efficiency (PCE) of Sb_2_S_3_ solar cells was increased from 4.26% to 5.15%, achieving the highest efficiency for Sb_2_S_3_ solar cells based on the CBD‐AT system. These results shed new light on fabricating efficient Antimony‐based solar cells by the CBD method without suffering from severe raw material waste.

## Graphical abstract

A novel PR strategy was proposed to improve CBD‐derived Sb_2_S_3_ film quality. It synergistically optimized the CBO of CdS/Sb_2_S_3_ interface and the back‐contact barrier, while the V_S2_ donor defect was converted into S_Sb2_ acceptor defect with lower concentration. Benefiting from the synergistic improvement of *J*_SC_ and FF, the optimized Sb_2_S_3_ solar cell achieved a champion PCE of 5.15%.

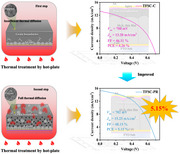

## 1 Introduction

In recent years, Sb_2_S_3_ attracted extensive research interest in fields of photovoltaic devices, photodetectors, indoor photovoltaics, and energy storage, etc. [1, 2, 3, 4, 5]. This is attributed to its remarkable merits, including high stability, simple composition, excellent resistance to radiation, a high theoretical power conversion efficiency (PCE) of 28%, etc. [6]. To date, despite the highest reported PCE of Sb_2_S_3_ TFSCs having been arrived at about 8.30% (certified PCE: 8.08%) [7, 8], this value still lags far behind its Shockley–Queisser (S–Q) limit, which primarily originates from the unsatisfactory quality of Sb_2_S_3_ absorber layer. Accordingly, developing reliable strategies to optimize absorber film quality and carrier transport behavior is imperative for further advancing the efficiency of Sb_2_S_3_ TFSCs.

Until now, various preparation methods have been exploited to increase the PCE of Sb_2_S_3_ thin film solar cells (TFSCs), including vacuum methods like vapor transporting deposition (VTD) [9], closed‐space sublimation (CSS) [10], and rapid thermal evaporation (ALD) [11], as well as solution methods like hydrothermal deposition (HTD) [7], chemical bath deposition (CBD) [12], spin‐coating method [13], etc. Among those methods, CBD stands out as one of the most promising routes for efficient Sb_2_S_3_ TFSCs due to its low‐temperature, atmospheric pressure, and large‐area deposition capability [12]. Currently, the CBD method for Sb_2_S_3_ thin films can be classified into two major systems according to the type of antimony source: the potassium antimony tartrate (PAT) system and the antimony trichloride (AT) system [12, 14]. For the PAT system, Wang et al. firstly proposed a multi‐sulfur source collaborative CBD approach to enhance the quality of Sb_2_S_3_ film in 2022, which enabled the PCE of Sb_2_S_3_ TFSCs to exceed 8% for the first time since 2014 [12, 15]. Subsequently, Shen et al. further increased the PCE of CBD‐derived Sb_2_S_3_ TFSCs to 8.26% by a strong chelating additive strategy [16]. Notably, the PAT‐based CBD method is quite similar to the widely adopted HTD method for Sb_2_S_3_ TFSCs, except that the former eliminates high‐temperature and high‐pressure deposition conditions and simplifies the process [16]. Hence, the plasticity of the precursor film will be greatly reduced, thereby suppressing the crystallinity of Sb_2_S_3_ film during the post‐annealing process [17]. Regarding the AT system, although it was developed the earliest, compared with the heterogeneous nucleation process of the PAT‐based CBD method, the AT‐based CBD method is prone to severe hydrolysis and homogeneous nucleation during deposition, inevitably inducing impurity phases and poor film morphology, as well as making the crystallization of the precursor film challenging [12, 15]. Fortunately, the quality of Sb_2_S_3_ films prepared by AT‐based CBD method can be significantly optimized by multiple strategies, including complexing agent engineering [18], regulation of S source [19], post‐annealing treatment [15], etc. For instance, Zhang et al. regulated the deposition kinetics of Sb_2_S_3_ precursor film by adding phosphotungstic acid in the AT‐based CBD process, thereby suppressing the formation of impurity phases, yielding a PCE of 4.61% [14]. B. Yang et al. modulated the nanoparticle formation‐adsorption pathway during the deposition of Sb_2_S_3_ precursor films, with sodium citrate employed as a complexing agent and thioacetamide serving as an additional S source, leading to a PCE of over 5.0% [18]. It is evident that both CBD systems are conducive to fabricating high‐quality Sb_2_S_3_ films. Nevertheless, Sb_2_S_3_ TFSCs derived from the AT‐based CBD process still require further optimization to boost device performance. Improving the crystallinity of Sb_2_S_3_ films stands as the key breakthrough for further boosting the PCE of Sb_2_S_3_ TFSCs [20]. Hence, exploring more efficient strategies to optimize film crystallinity is of great research significance. Meanwhile, both the solution methods and vacuum methods are suffering from inherently low raw material utilization, which severely limits their large‐scale industrial deployment. Especially for the fabrication of materials containing heavy metal elements (e.g., Ga, In, Cd, etc.), potential environmental pollution and associated hazards are also nonnegligible concerns, thereby leading to an increase in the preparation cost in turn [21]. Exploring strategies to simultaneously improve the raw material utilization efficiency and device performance of Sb_2_S_3_ TFSCs is critical for enhancing the competitiveness of the solution methods in the future industrialization arena.

In this work, to boost the PCE of CBD‐derived Sb_2_S_3_ TFSCs and enhance raw material utilization efficiency during deposition, an innovative precipitate recycling (PR) strategy of CBD‐generated Sb_2_S_3_ precipitates was proposed to tailor crystallization kinetics via modulating the microstructure of the precursor films. Specifically, Sb^3+^ cations possess unique 5 s^2^ orbitals and can form stable coordination complexes to modulate their release behavior. The as‐prepared Sb_2_S_3_ precursor film by CBD method was subsequently treated in an NH_3_‐EG mixed solution that the Sb_2_S_3_ precipitates were dissolved in it. This recycling strategy achieves high‐efficiency recycling of the precipitates, and endows the Sb_2_S_3_ precursor film with a porous structure while maintaining its compactness. Accordingly, the dissolving mechanism of Sb_2_S_3_ precipitates in NH_3_‐EG mixed solution was well studied. The differences in deposition behavior of the Sb_2_S_3_ precursor films before and after modification of PR strategy were systematically investigated. As a result, the crystallization quality and the [*hk*1] orientation growth of Sb_2_S_3_ film were greatly enhanced, and the nonradiative carrier recombination loss in Sb_2_S_3_ TFSCs was effectively suppressed. Interestingly, the CBO of CdS/Sb_2_S_3_ interface was optimized to −0.17 eV, the back barrier was reduced by 35%, and the V_S2_ donor defect was converted into S_Sb2_ acceptor defect, accompanied by a nearly one‐order‐of‐magnitude reduction in defect concentration. Finally, benefiting from the synergistic optimization of *J*_SC_ and FF, the PCE of Sb_2_S_3_ TFSCs was obviously increased from 4.26% to 5.15%. The findings can offer valuable guidance for fabricating efficient Sb_2_S_3_ TFSCs and improve the industrial competitiveness of CBD method in thin film solar cells.

## 2 Results and Discussion

**FIGURE 1:**
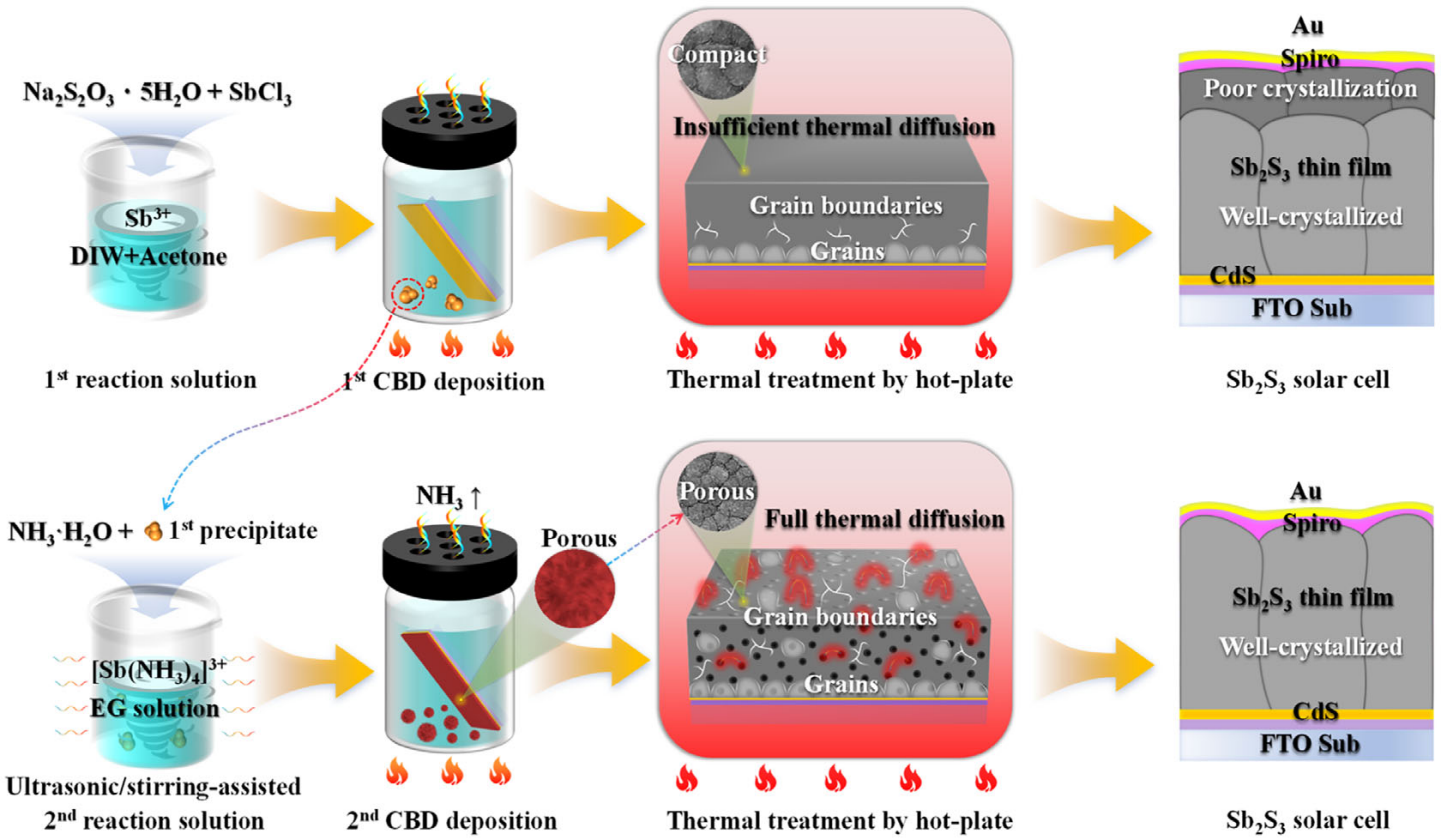
Schematic diagram of Sb_2_S_3_ thin films and solar cells prepared by AT‐based CBD process without and with modification of PR strategy.

As shown in Figure 1, for the conventional AT‐based CBD process, the film deposition is driven by an ion‐by‐ion condensation mechanism interwoven with the transient complex decomposition. Specifically, Sb^3+^ cations interact with thiosulfate ions to form an antimony thiosulfate complex intermediate [14]. Meanwhile, uncoordinated thiosulfate ions undergo continuous hydrolytic decomposition and gradually release highly reactive sulfide (S^2−^) species into the reaction solution [14]. Once the ionic product (IP) of free Sb^3+^ and S^2−^ ions steeply surpasses the solubility product constant of Sb_2_S_3_, an overwhelming thermodynamic driving force is instantly generated to precipitate solid Sb_2_S_3_ [22]. The fundamental deposition process can be described as the following equation:

(1) $$2\mathrm{SbC}l_{3}+3Na_{2}S_{2}O_{3}+3H_{2}O=Sb_{2}S_{3}+3Na_{2}SO_{4}+6\mathrm{HCl}$$

It is well known that a well‐deposited Sb_2_S_3_ precursor film by CBD method is amorphous and compact. Such compact morphology limits the subsequent thermal diffusion during post‐annealing, hindering the full crystallization of Sb_2_S_3_ film and inducing inhomogeneous crystallization along the film thickness. This ultimately degrades carrier transport properties and reduces the PCE of Sb_2_S_3_ TFSCs. In addition, low raw material utilization has long been one of the major drawbacks of solution methods compared with vacuum methods. The conventional CBD method is plagued by an extremely low atom utilization efficiency, typically below 10% [23]. To address the above issue, an innovative PR strategy of CBD‐generated Sb_2_S_3_ precipitates is proposed to optimize the morphology of Sb_2_S_3_ precursor film. Benefiting from the unique 5 s^2^ orbitals of Sb^3+^, which enable the formation of stable coordination complexes with additives, an NH_3_‐EG mixed solution is adopted to dissolve the Sb_2_S_3_ precipitates collected from the AT‐based CBD process in this work. EG possesses two hydroxyl groups (‐OH) and exhibits high viscosity, low surface tension, as well as bidentate ligand characteristics. Under alkaline conditions, EG can be deprotonated to form ‐OCH_2_CH_2_O‐ (glycolate, EG^2−^) anion, thereby forming strong coordination with Sb^3+^ ions to generate soluble antimony‐glycolate chelate anions [24, 25]. Moreover, as EG is a polar aprotic solvent (relative to water), it can inhibit the hydrolysis of S^2−^ ions, resulting in the S^2−^ ions mainly existing as HS^−^ ions or unstable (NH_4_)_2_S [26]. During the subsequent deposition process, with the volatilization of NH_3_ gas, the S^2−^ ions and Sb^3+^ ions will be slowly released from HS^−^ ions (or unstable (NH_4_)_2_S) and glycolate ligands, respectively. Leading to the re‐formation of Sb_2_S_3_. Notably, due to the volatilization of ammonia, the originally dense precursor film will form a porous morphology without damaging the compactness. Such a porous morphology can bring the following benefits for Sb_2_S_3_ film during the subsequent annealing process: (1) promoting thermal diffusion; (2) facilitating elemental migration; 3) relieving internal stress. Hence, after modification with the PR strategy, the crystallization of Sb_2_S_3_ film is greatly enhanced, and the carrier transport ability will be optimized as well, particularly in the rear region of Sb_2_S_3_ film.

**FIGURE 2:**
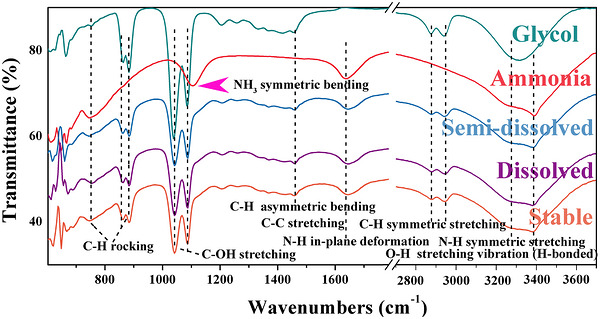
FTIR spectra of glycol, ammonia, semi‐dissolved, dissolved, and stable Sb_2_S_3_ precursor solutions.

As shown in Figure S1, three distinct dissolution planes of Sb_2_S_3_ precipitates are explored under ultrasonic treatment. Routes 1 and 2 demonstrate that although aqueous ammonia can dissolve Sb_2_S_3_ precipitates by forming [Sb(NH_3_)_4_]^3+^ coordination complexes, its dissolving capacity is limited. Moreover, it readily induces the hydrolysis of Sb^3+^ ions, accompanied by the generation of white precipitates such as Sb_2_O_3_ or Sb(OH)_3_. By contrast, the introduction of ethylene glycol (EG) effectively stabilizes the solution system and yields a transparent precursor solution. Finally, the optimal dissolution ratio was determined as 0.05 g Sb_2_S_3_ precipitates dissolved in a mixed solution of 10 mL EG and 9 mL ammonia. Meanwhile, with the increase in the content of Sb_2_S_3_ precipitates, the transparent precursor solution gradually changes from colorless to brick‐red (Figure S2). According to the Fourier Transform Infrared Spectroscopy (FTIR) spectra of different solvents and precursor solutions presented in Figure 2, compared with the FTIR spectra of aqueous ammonia and EG, the characteristic vibration peak of NH_3_ disappears in the precursor solution, and the C─OH stretching peak is remarkably weakened. This indicates that NH_3_ and ─OH groups act as ligands and participate in the dissolution of Sb_2_S_3_ precipitates. In addition, although the solution gradually changes from brick red to colorless and transparent with prolonged standing, no significant variation is observed in its FTIR spectra. Therefore, at the initial dissolution stage, Sb_2_S_3_ precipitates will react with aqueous ammonia to form [Sb(NH_3_)_4_]^3+^ coordination complexes (Equation 2) [27], which strongly absorb visible light and impart a brick‐red color to the solution. In this process, EG serves as a stabilizer to suppress the hydrolysis of Sb^3+^ ions and maintain the stability of [Sb(NH_3_)_4_]^3+^ coordination complexes. With prolonged standing, EG gradually replaces NH_3_ ligands in [Sb(NH_3_)_4_]^3+^ coordination complexes via its strong complexing capability to form Sb‐EG chelates with monodentate coordination (Equation 3) [25]. As the standing time further increases, the unstable Sb‐EG chelates evolve into cyclic chelates through bidentate coordination (Equation 4) [25]. At this stage, the valence state of the chelates decreases, the solution becomes stable gradually, and its color fades to orange‐yellow. Eventually, EG completely replaces the NH_3_ ligand inside the chelates under high pH conditions to form Sb(OCH_2_CH_2_O)_2_ dimers, yielding a stable, colorless, and transparent precursor solution.

(2) $${\left[\mathrm{Sb}{\left(NH_{3}\right)}_{4}\right]}^{3+}+\mathrm{EG}\;\overset{\mathrm{Storage}}{\Leftrightarrow }{\left[\mathrm{Sb}{\left(NH_{3}\right)}_{3}\left(\mathrm{EG}\right)\right]}^{3+}+NH_{3}↑$$

(3) $${\left[Sb{\left(NH_{3}\right)}_{3}\left(EG\right)\right]}^{3+}\;\overset{Storage}{\Leftrightarrow }{\left[Sb{\left(NH_{3}\right)}_{2}\left(OCH_{2}CH_{2}O\right)\right]}^{+}+2NH_{3}↑$$

(4) $${\left[Sb{\left(NH_{3}\right)}_{2}\left(OCH_{2}CH_{2}O\right)\right]}^{+}+EG\;\overset{Storage}{\Leftrightarrow }Sb{\left(OCH_{2}CH_{2}O\right)}_{2}+2NH_{3}↑$$

To prove the effect of the obtained precursor solution in preparing Sb_2_S_3_ films by the CBD process and the reusability of the Sb_2_S_3_ precipitates, the Sb_2_S_3_ films are deposited using the precursor solution obtained through Sb_2_S_3_ precipitates collected from different deposition cycles. As presented in Figure S3, the collected first‐cycle Sb_2_S_3_ precipitates exhibit a quasi‐amorphous structure. The presence of considerable Sb_2_O_3_ secondary phases agrees well with the inherent hydrolysis tendency of the CBD process based on AT system. This finding can be further evidenced by the Raman peak (298 cm^−1^) of Sb‐O band vibrations in Figure S4a. Given the insolubility of Sb_2_O_3_ in weak alkaline solution, the secondary‐cycle Sb_2_S_3_ precipitate is pure‐phase Sb_2_S_3_. Moreover, the morphology of the films and sediments obtained in the second and third cycles differs greatly from that of the first cycle, presenting a loose, porous morphology with favorable continuity. From the BET specific surface area and pore size distribution results of the first‐ and second‐collected Sb_2_S_3_ precipitates in Figure S5a,b, compared with the spherical precipitates formed by conventional CBD deposition, the specific surface area of the flower‐like precipitates obtained after PR strategy increased by nearly 19 times, and the volume of the holes has also significantly expanded. Notably, Sb and S elements are uniformly distributed in the first‐ and second‐collected Sb_2_S_3_ precipitates, both of which exhibit an S‐rich composition, and the latter are richer in sulfur (Figure S6). This is beneficial for the subsequent preparation of high‐quality Sb_2_S_3_ films. That is because EG functions as a bidentate chelating ligand and adsorbs on the surface of Sb(OCH_2_CH_2_O)_2_ complexes during film deposition process. The resultant strong steric hindrance suppresses the close growth and densified aggregation of Sb_2_S_3_ grains. Meanwhile, the release of NH_3_ generates microbubbles throughout the deposition process. Combined effects of the above two factors render the as‐prepared film a porous structure. To evaluate the film quality, the Sb_2_S_3_ thin films obtained from the first, second, and third cycles are assembled into complete Sb_2_S_3_ TFSCs, denoted as TFSC‐C, TFSC‐R1, and TFSC‐R2, respectively. The device performances of TFSC‐R1 and TFSC‐R2 devices are slightly inferior to that of TFSC‐C device (Figure S4b). This is mainly attributed to the deteriorated CdS/Sb_2_S_3_ interfacial quality caused by the etching effect of NH_3_‐EG mixed solution, which can be demonstrated by the reduced external quantum efficiency (EQE) response of TFSC‐R1 and TFSC‐R2 devices in the range of below 520 nm (Figure S4c). These results demonstrate the feasibility of this precipitate recycling strategy and its potential for unlimited recycling.

**FIGURE 3:**
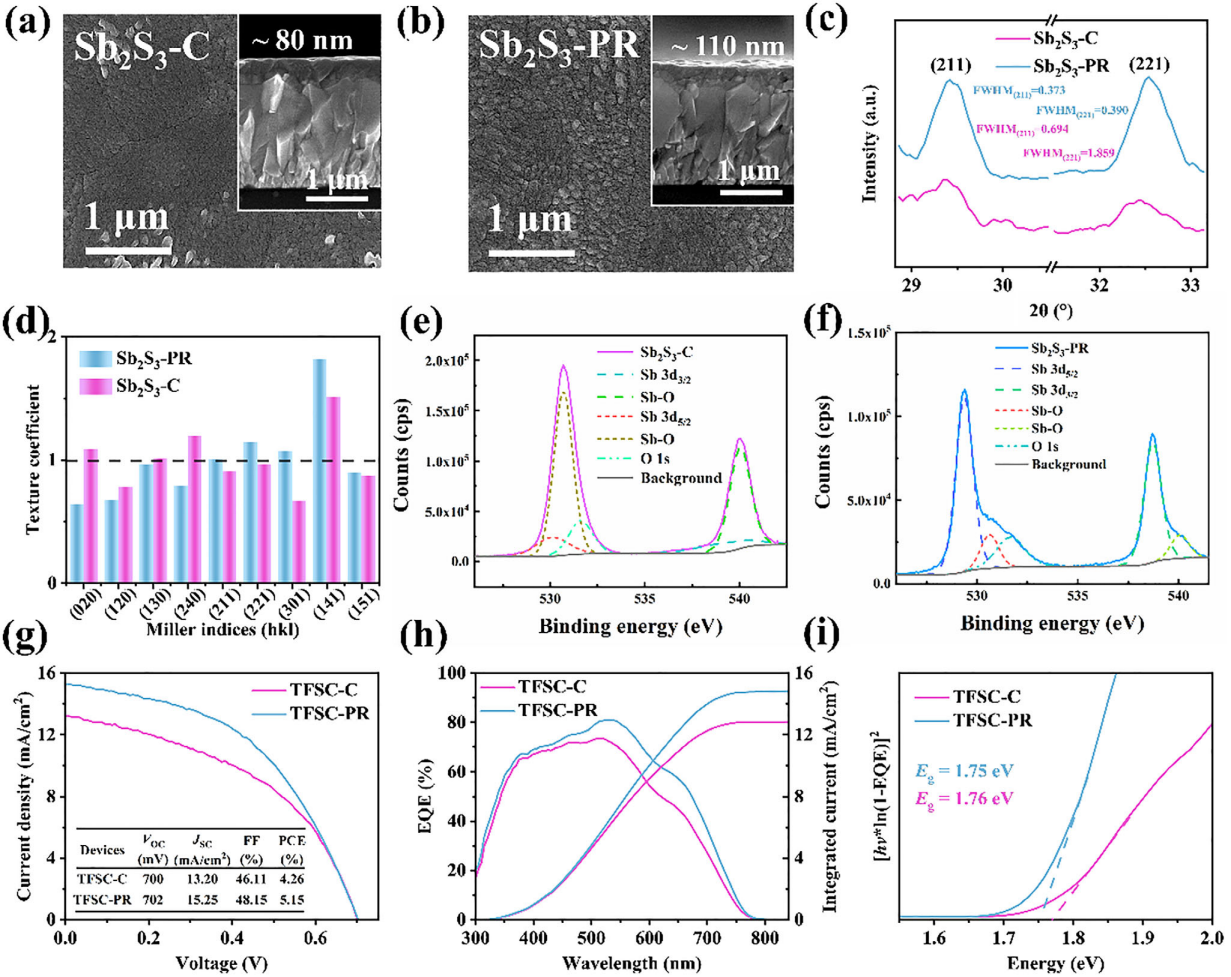
SEM images of (a) Sb_2_S_3_‐C and (b) Sb_2_S_3_‐PR films; (c) enlarged XRD patterns in 29°–33° and (d) TC values of the selected planes for the two Sb_2_S_3_ films; XPS analysis of the Sb_2_S_3_ films: Sb 3d core levels of (e) Sb_2_S_3_‐C and (f) Sb_2_S_3_‐PR films. (g) *J*–*V* curves, (h) EQE spectra and (i) the extracted *E*_g_ values for TFSC‐C and TFSC‐PR devices.

To improve the crystallization ability of Sb_2_S_3_ precursor films fabricated by the conventional CBD method, the as‐prepared Sb_2_S_3_ precursor film is deposited in the precursor solution derived from recycled Sb_2_S_3_ precipitates for morphological modification. Prior to that, the quality of Sb_2_S_3_ thin films prepared by the conventional one‐step CBD process is investigated by adjusting the deposition time. As shown in Figure S7a and Table S1, the Sb_2_S_3_ TFSCs achieve an optimal efficiency of 4.26% at a deposition time of 55 min, with a corresponding absorber thickness of 80 nm (Figure 3a). Subsequently, the primary precursor film is subjected to secondary deposition in the recycled Sb_2_S_3_ precipitates‐derived precursor solution to modify its morphology, and the secondary deposition time is further optimized. As displayed in Figure S7b and Table S2, the modified Sb_2_S_3_ TFSCs yield a champion efficiency of 5.15% at a secondary deposition time of 15 min, with an absorber thickness of 110 nm (Figure 3b). Therefore, to further evaluate the performance enhancement brought by the precipitate recycling (PR) strategy, the optimal Sb_2_S_3_ films and corresponding devices prepared by the one‐step and two‐step routes are systematically compared to reveal the advantages of this strategy. For better clarity, the Sb_2_S_3_ films and solar cells prepared by the one‐step and two‐step methods are named as Sb_2_S_3_‐C/Sb_2_S_3_‐PR and TFSC‐C/TFSC‐PR, respectively. The precursor films before and after the PR strategy are denoted as Precursor‐C and Precursor‐PR, respectively. As presented in Figure S8a,b, the compact morphology of the Sb_2_S_3_ precursor film changes significantly after the PR strategy. Compared with the Precursor‐C film, the Precursor‐PR film displays a more porous morphology and improved crystallinity. In addition, the thickness of the Sb_2_S_3_ precursor film increases from 90 to 125 nm, suggesting that the PR strategy can not only tailor the film morphology, but also effectively increase its thickness. Therefore, owing to the porous morphology induced by PR strategy, the Sb_2_S_3_‐PR film exhibits better crystallinity than Sb_2_S_3_‐C films after the post‐annealing process (Figure 3a,b). According to the grazing incidence X‐ray diffraction (GIXRD) patterns shown in Figure S9, the Sb_2_S_3_‐PR film exhibits superior crystallinity compared with Sb_2_S_3_‐C film. The obviously reduced full width at half maximum (FWHM) of the (211) and (221) diffraction planes for Sb_2_S_3_‐PR film further confirms its enhanced crystallinity (Figure 3c). It is worth noting that the preferred [*hk*0] orientation of the Sb_2_S_3_ film is weakened, while the [*hk*1] orientation is enhanced (Figure 3d). Additionally, as the X‐ray photoelectron spectroscopy (XPS) results of the two films in Figure 3e,f and Figure S10, compared with the Sb_2_S_3_‐C film, the Sb_2_S_3_‐PR film shows higher intensity of the Sb 3d_5/2_ and Sb 3d_3/2_ XPS peaks and reduced XPS peaks intensity of Sb‐O bonds, which confirms that the PR strategy successfully mitigates the oxidation of the Sb_2_S_3_ film [28]. Meanwhile, in addition to the XPS signals of lattice S (S 2p_1/2_‐A and S 2_p3/2_‐A), peaks corresponding to oxidized sulfur species are also observed (S 2p_1/2_‐B and S 2_p3/2_‐B) [29]. Upon adopting the PR strategy, the XPS peak intensity of lattice sulfur is enhanced, whereas that of oxidized sulfur is markedly diminished. This finding further demonstrates the suppressed oxidation in Sb_2_S_3_‐PR films. Consequently, the TFSC‐PR device achieves a better performance than TFSC‐C device due to the greatly enhanced *J*_SC_ and FF (Figure 3g). The improved *J*_SC_ can be attributed to the optimized [*hk*1] orientation and enhanced crystallinity of Sb_2_S_3_ film after PR treatment, while the improved FF originates from the promoted carrier transport capability. Besides, the increased thickness of Sb_2_S_3_‐PR film enhances the absorption of TFSC‐PR device toward incident light in the range of 300–700 nm (Figure S11a), which also contributes to the enhancement of *J*_SC_. Thus, the TFSC‐PR device exhibits a remarkably enhanced EQE response in the wavelength range of 500–700 nm compared with TFSC‐C device (Figure 3h). As depicted in Figure 3i and Figure S11b, owing to the Sb_2_S_3_‐PR film possessing a higher sulfur content than the Sb_2_S_3_‐C film, the TFSC‐PR device exhibits a slightly narrower bandgap energy (*E*_g_) of 1.75 eV compared with that of TFSC‐C device (1.76 eV), suggesting a reduced density of sulfur vacancy (V_S_) defects in the former. As shown in Figure S12a‐d and Table S3, statistical performance analysis of 25 individual devices further verifies that the PR strategy can effectively improve *J*_SC_ and FF, thereby demonstrating the good repeatability and reliability of this modification strategy. To further clarify the influence of the PR strategy on device performance, Figure S13a‐d shows that the TFSC‐PR device delivers a much higher shunt resistance (*R*_sh_) of 854.70 Ω·cm^2^ than TFSC‐C device (16.67 Ω·cm^2^), while the series resistance (*R*_s_) declines from 8.18 to 3.97 Ω·cm^2^. These variations reflect the improved film compactness and enhanced carrier transportation ability of Sb_2_S_3_‐PR film. After PR treatment, the *A* factor decreases from 3.22 to 1.98, implying suppressed trap‐assisted carrier recombination in the bulk of Sb_2_S_3_ film. Meanwhile, the decreased saturation current density (*J*_0_), from 1.78×10^−4^ mA/cm^2^ to 1.81×10^−6^ mA/cm^2^, further confirms that the TFSC‐PR device has superior film quality and mitigated recombination loss.

**FIGURE 4:**
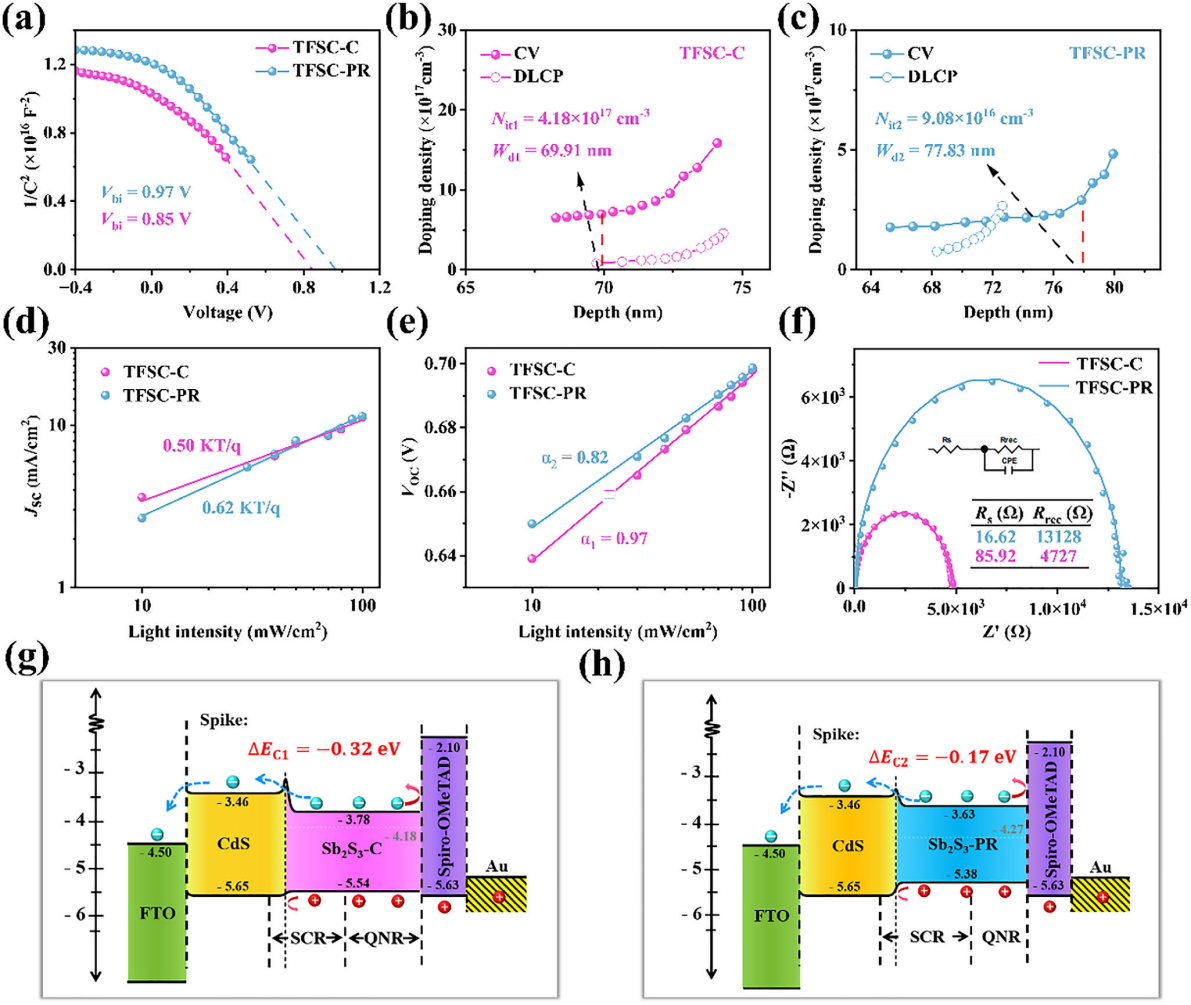
(a) 1/*C* ^2^‐*V* plots, (b/c) *C*‐*V* & DLCP curves and light‐intensity dependent (d) *J*_SC_ and (e) *V*_OC_ curves for TFSC‐C and TFSC‐PR devices; (f) Nyquist EIS curves for TFSC‐C and TFSC‐PR devices; Bandgap alignment diagrams (g) of TFSC‐C and (h) TFSC‐PR devices.

To further elucidate the performance differences induced by the PR strategy, the electrical characteristics of TFSC‐C and TFSC‐PR devices are systematically investigated and presented in Figure 4. It is found that TFSC‐PR device has a larger *V*_bi_ (0.97 V) compared to TFSC‐C device (0.85 V), which demonstrates the better quality of CdS/Sb_2_S_3_‐PR heterojunction than CdS/Sb_2_S_3_‐C heterojunction (Figure 4a). As depicted in Figure 4b,c, the concentration of interfacial defects in TFSC‐PR device (9.08 × 10^16^ cm^−3^) is significantly decreased compared to that of TFSC‐C device (4.18 × 10^17^ cm^−3^). It reveals that the PR strategy can effectively optimized the quality of the rear region in Sb_2_S_3_ TFSCs by enhancing crystallization and [*hk*1] orientation, leading to an increase in *J*_SC_ and FF. In addition, the wider depletion region (*W*_d2_ = 77.83 nm) of TFSC‐PR device is larger than that of TFSC‐C device (*W*_d1_ = 69.91 nm). In view of the thickness of Sb_2_S_3_‐PR film (∼110 nm) is larger than Sb_2_S_3_‐C film (∼80 nm), the larger width of quasi‐neutral region for TFSC‐PR device (∼32 nm) compared with TFSC‐C device (∼10 nm) indicates the better carrier transport in Sb_2_S_3_‐PR film. Moreover, the light intensity‐dependent *J*_SC_ and *V*_OC_ characteristics (Figure 4d,e) of the two devices are analyzed by Equations (5) and (6) [30, 31]:

(5) $$V_{\mathrm{OC}}=\frac{\mathrm{nKT}}{q}\ln\left(\frac{I_{0}}{I}+1\right)$$

(6) $$J_{\mathrm{SC}}\propto I^{\alpha }\left(\alpha \leq 1\right)$$

where *n* is the ideality factor, *q* is the elementary charge, *k* is the Boltzmann constant, *I*_0_ denotes the initial light intensity, *T* is the absolute temperature, and *α* represents the correlation coefficient between *J*_SC_ and light intensity. The *α* value of the TFSC‐PR device (*α*_2_ = 0.97) is higher than that of the TFSC‐C device (*α*_1_ = 0.82), demonstrating the improved carrier transport capability of the TFSC‐PR device. Meanwhile, the reduction in *n* value from 0.62 to 0.50 reveals effective suppression of trap‐assisted carrier recombination in the TFSC‐PR device. According to the transient photocurrent (TPC) and transient photovoltage (TPV) results presented in Figure S14a,b, the average transporting lifetime of TFSC‐PR device (τ_t2_ = 55.64 µs) is shorter than that of TFSC‐C device (τ_t1_ = 85.74 µs), while the average recombination lifetime of TFSC‐PR device (τ_r2_ = 0.39 ms) is extended in comparison with that of TFSC‐C device (τ_r1_ = 0.12 ms). It indicates that carriers in Sb_2_S_3_ TFSCs transport faster and suffer from less trap‐assisted recombination after PR strategy. Moreover, as displayed in Figure S14c and Figure 4f, compared with TFSC‐C device, the Urbach energy (*E*_u_) of TFSC‐PR device is reduced from 26.61 meV to 24.20 meV. Meanwhile, the series resistance (*R*_s_) declines from 13.83 Ω to 13.24 Ω, while the recombination resistance (*R*_rec_) rises markedly from 2529 Ω to 8684 Ω. According to the Ultraviolet Photoelectron Spectroscopy (UPS) results shown in Figure S15 and Equation (7):

(7) $$\varphi =h\nu -E_{cutoff}$$

where *hν* is the energy of the ultraviolet electron (21.22 eV). The Fermi energy levels are calculated as −4.18 eV for Sb_2_S_3_‐C film (with valence band maximum (*V*_BM_) of −5.54 eV) and −4.27 eV for Sb_2_S_3_‐PR film (with *V*_BM_ of −5.38 eV). Subsequently, according to the former bandgap (*E*_g_) results, the conduction band minimum (*C*_BM_) of Sb_2_S_3_‐C and Sb_2_S_3_‐PR films are calculated as −3.78 and −3.63 eV, respectively. It is not difficult to conclude that both Sb_2_S_3_‐C and Sb_2_S_3_‐PR films exhibit N‐type characteristics. Furthermore, the corresponding bandgap alignments of the two devices are illustrated in Figure 4g,h, where the bandgap structures of Spiro‐OMeTAD and CdS are consistent with the previous literature [32]. Significantly, the CdS/Sb_2_S_3_‐PR interface exhibits a larger conduction band offset (CBO) (Δ*E*_c2_ = −0.17 eV) compared with that of CdS/Sb_2_S_3_‐C interface (Δ*E*_c1_ = −0.32 eV), suggesting enhanced interfacial carrier transport in TFSC‐PR device.

**FIGURE 5:**
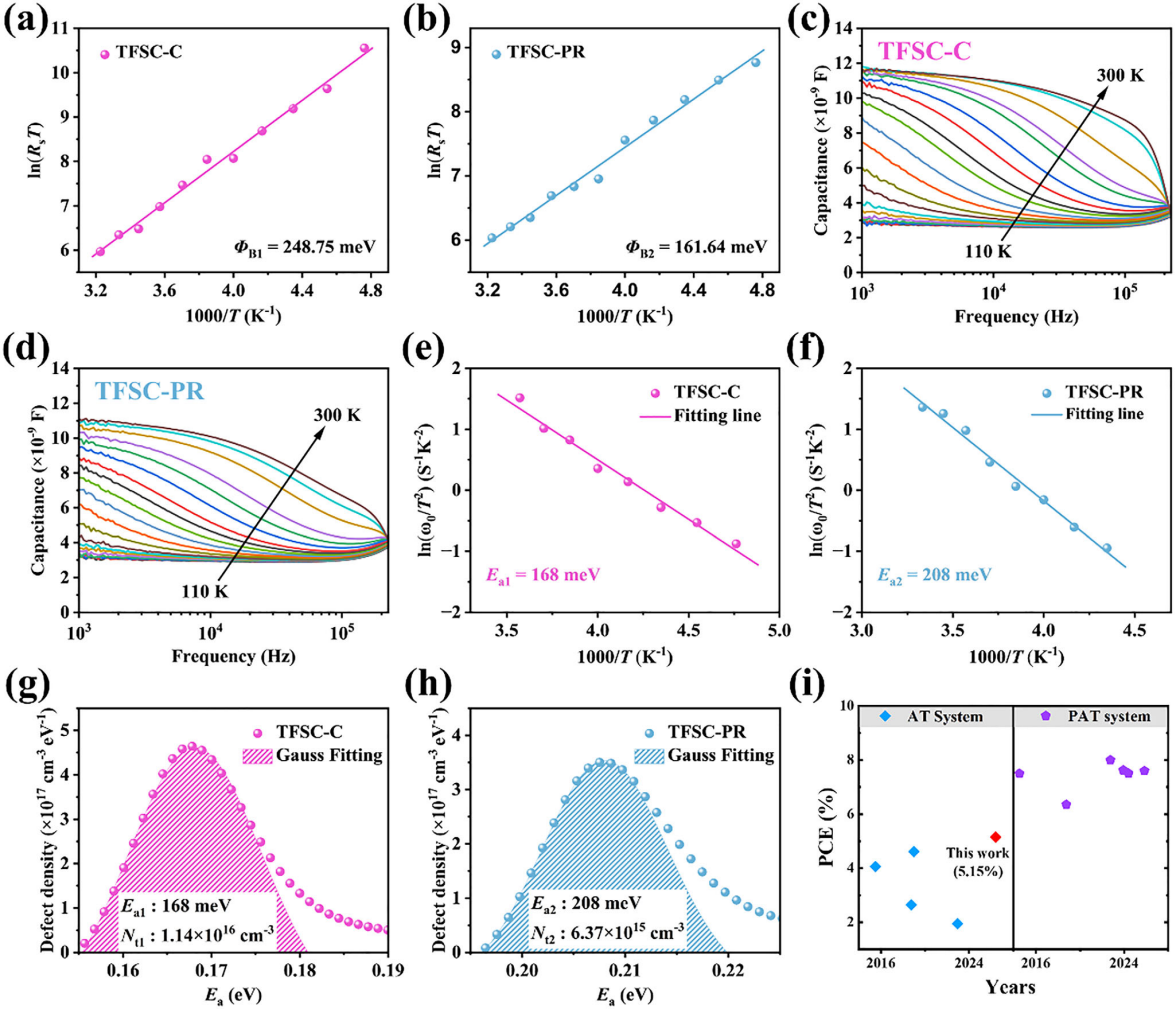
ln(*R*_s_ *T*) vs. 1000/*T* plots of (a) TFSC‐C and (b) TFSC‐PR devices; (c/d) Temperature‐dependent admittance spectroscopy, (e/f) Arrhenius plots of ln(ω_0_/*T* ^2^) versus 1000/*T* and (g/h) defect densities for TFSC‐C/TFSC‐PR device; (i) PCE statistical analysis of planar heterojunction Sb_2_S_3_ TFSCs processed by CBD method.

The back‐contact barrier height (*Φ*_B_) of the solar cell represents the difficulty of extracting holes from the back interface. To quantify the influence of the PR strategy on the back interface of Sb_2_S_3_ TFSCs, the *Φ*_B_ of the two devices are extracted from temperature‐dependent dark *J*‐*V* curves presented in Figure S16 according to Equation (7) [33]:

(8) $$R_{s}=\frac{K}{qA^{*}}exp\left(\frac{\Phi_{B}}{kT}\right)$$

where *K* is the Boltzmann constant, *A* ^*^ is the effective Richardson constant, *q* is the electron charge, and *R*_s_ represents the temperature‐dependent series resistance. As shown in Figure 5a,b, the *Φ*_B_ of TFSC‐PR device (161.64 meV) is obviously lower than that of TFSC‐C device (248.75 meV). This can be ascribed to the optimized crystallinity and [*hk*1] preferred orientation of Sb_2_S_3_ film. Accordingly, combined with the reduction in the CBO value of CdS/Sb_2_S_3_ interface, these results fully demonstrate that the PR strategy can effectively suppress the nonradiative carrier recombination in CBD‐processed Sb_2_S_3_ TFSCs, and synergistically improve carrier transport at both the front and back interfaces of TFSC‐PR devices.

To further elucidate the defect characteristics of the two devices, as shown in Figure 5c,d, according to Equation (8) [34]:

(9) $$\omega_{0}=2\pi v_{0}exp\frac{-E_{a}}{KT}$$

where *ν*_0_ is the electron escape frequency attempt value, *K* is the Boltzmann constant. It is found that defect D_1_ with an activation energy (*E*_a1_) of 168 meV is detected in TFSC‐C device, whereas the corresponding activation energy (*E*_a2_) of defect D_2_ in TFSC‐PR device is determined to be 208 meV (Figure 5e,f). Furthermore, the defect density (*N*_t_) is further calculated according to Equations (9) and (10) [34]:

(10) $$E\;\left(\omega \right)=kTln\left(\frac{2\pi v_{0}T^{2}}{\omega }\right)$$

(11) $$N_{t}\left(E\left(\omega \right)\right)=-\frac{V_{d}}{q\omega }\frac{dC}{d\omega }\frac{\omega }{kT}$$

where *V*_d_ is the built‐in potential of the p‐n junction, and *N*_t_(E(ω)) represents the defect density. As shown in Figure 5g,h, the defect density of the D_2_ defect in TFSC‐PR device is 6.37 × 10^15^ cm^−3^, markedly lower than 1.14 × 10^16^ cm^−3^ for D_1_ defect in TFSC‐C device. Given the higher S content in Sb_2_S_3_‐PR film compared with Sb_2_S_3_‐C film (Figure S5), combined with previous first‐principles calculations of Sb_2_S_3_ [35] and the characteristics of S loss during the annealing process [7, 34], the D_1_ defect can be defined as V_S2_ donor defect owing to its lowest formation energy. This dominant defect largely governs the electrical conductivity of Sb_2_S_3_‐C film, and consequently constrains the *J*_SC_ and FF of TFSC‐C device. On the other hand, the D_2_ defect can be defined as S_Sb2_ acceptor defect rather than O_S_ and O_Sb_ defects, as S_Sb2_ defect possesses a lower formation energy and a higher equilibrium concentration than the latter two. Accordingly, the Sb_2_S_3_‐PR film exhibits stronger p‐type conductivity than the Sb_2_S_3_‐C film, contributing to the remarkable improvement in the *J*_SC_ and FF of TFSC‐PR device. To better describe the defect properties of both devices, the capture cross‐section (*σ*_0_) and corresponding capture lifetime (*τ*) of defect states are further calculated by Equations (11) and (12) [36]:

(12) $$\sigma_{0}=\frac{\omega_{0}}{2N_{c,v}v_{th}exp\left(\frac{-E_{a}}{KT}\right)}$$

(13) $$\tau =\frac{1}{\sigma_{0}v_{th}N_{t}}$$

where *v*_th_ is the thermal velocity, and *σ*_0_ is extracted by fitting the admittance spectroscopy curves. As shown in Table 1, the *σ*_0_ value of TFSC‐PR device decreases notably to 4.68 × 10^−17^ cm^2^ by comparing with that of 6.27 × 10^−17^ cm^2^ for TFSC‐C device. Meanwhile, the τ value of TFSC‐PR device is markedly extended to 0.33 µs, in contrast to 0.14 µs for the TFSC‐C device. More importantly, the product of trap density and capture cross‐section (*N*_t_×*σ*_0_), a critical metric characterizing trap‐assisted recombination, declines substantially from 7.15 × 10^−1^ cm^−1^ in the TFSC‐C device to 2.98 × 10^−1^ cm^−1^ in the TFSC‐PR device. These results demonstrate that the PR strategy can effectively transform V_S2_ donor defects into S_Sb2_ acceptor defect, while greatly suppressing defect density and mitigating nonradiative carrier recombination loss. Herein, benefiting from the enhanced *J*_SC_ and FF, the TFSC‐PR device achieves a champion PCE of 5.15%. This efficiency represents the state‐of‐the‐art performance of Sb_2_S_3_ TFSCs fabricated via the AT‐based CBD method (Figure 5i).

**TABLE 1:** Conductivity characteristic parameters of Sb_2_S_3_ devices.

Devices	*E*_a_ (eV)	*N*_t_ (cm^−3^)	*σ*_0_ (cm^2^)	*τ* (µs)	*N*_t_ ×*σ*_0_ (cm^−1^)
TFSC‐C	0.168	1.14 × 10^16^	6.27 × 10^−17^	0.14	7.15 × 10^−1^
TFSC‐PR	0.208	6.37 × 10^15^	4.68 × 10^−17^	0.33	2.98 × 10^−1^

## 3 Conclusion

In summary, to further boost the PCE of CBD‐derived Sb_2_S_3_ TFSCs and improve raw material utilization during deposition, an innovative precipitate recycling (PR) strategy was proposed for CBD‐generated Sb_2_S_3_ precipitates. The advantages of the PR strategy proposed in this work can be summarized as follows: (1) the NH_3_‐EG solution effectively dissolves Sb_2_S_3_ precipitates produced during the CBD process, achieving high‐efficiency recycling of raw materials; (2) Enabling the Sb_2_S_3_ precursor film a porous morphology by secondary deposition process, providing sufficient spatial freedom for thermal diffusion and elemental migration during annealing; (3) Improving the crystallinity and [*hk*1] preferred orientation of Sb_2_S_3_ films, and leading to enhanced carrier transport in Sb_2_S_3_ solar cells via optimizing the CBO of CdS/Sb_2_S_3_ interface from −0.32 to −0.17 eV, reducing the interfacial defect density by 3.27 × 10^16^ cm^−3^ and the back‐contact barrier by 35%; (4) Converting V_S2_ donor defect into S_Sb2_ acceptor defect, accompanied by a nearly one‐order‐of‐magnitude reduction in defect concentration. Consequently, benefiting from the synergistic improvement of *J*_SC_ and FF, the PCE of CBD‐processed Sb_2_S_3_ TFSCs was significantly improved from 4.26% to 5.15%. Notably, the champion PCE achieved in this work represents the state‐of‐the‐art performance for Sb_2_S_3_ TFSCs fabricated via the antimony trichloride (AT)‐based CBD method, highlighting the effectiveness and superiority of the proposed PR strategy. Nevertheless, compared to state‐of‐the‐art Sb_2_S_3_ solar cells [37], the high density of grain boundaries and insufficient light absorption are the two main factors that limit the PCE of Sb_2_S_3_ solar cells from further improvement. Therefore, optimizing the annealing conditions and absorber thickness is the key to further enhance the performance of CBD‐derived Sb_2_S_3_ TFSCs based on AT system.

## 4 Experimental Section

### 4.1 Preparation of the Precursor Solutions for Depositing

FTO substrates were ultrasonically cleaned sequentially with detergent, acetone, anhydrous ethanol, and deionized water for 35 min each. Then, a 60 nm thick CdS electron transporting layer was deposited on the cleaned FTO substrates and then treated with CdCl_2_ methyl alcohol solution following the previous report [38]. For the precursor solution used for conventional CBD deposition of Sb_2_S_3_ film, first, 5 mL of 0.7 M SbCl_3_ acetone solution, 25 mL of 1 M Na_2_S_2_O_3_·5H_2_O aqueous solution, and 75 mL of DI water were separately prepared and cooled to 0°C. Afterward, the above solutions were mixed to form the initial CBD reaction solution for Sb_2_S_3_ deposition (Solution A). For the recycling strategy of CBD‐derived sediments, the Sb_2_S_3_ sediments were first collected from the waste reaction solution of conventional CBD processes. The harvested sediments were thoroughly rinsed with anhydrous ethanol and dried for subsequent use. Next, 0.04 g Sb_2_S_3_ sediments were dispersed into a mixed solvent consisting of ammonia solution and ethylene glycol at a volume ratio of 9:10. The resulting mixture was then subjected to ultrasonic treatment and magnetically stirred at room temperature until a homogeneous, clear solution was obtained (Solution B).

### 4.2 Deposition of Sb_2_S_3_ Films

For comparison, the pristine Sb_2_S_3_ film (denoted as Sb_2_S_3_‐C) was fabricated via a conventional CBD route. Briefly, FTO/CdS substrates were immersed in Solution A and maintained at 25°C for 55 min. After reaction completion, the substrates were retrieved, rinsed with deionized water, and dried. The obtained Precursor‐C films were subsequently heated at 360°C for 5 min and 160°C for 1 h under a nitrogen atmosphere to obtain a crystallized Sb_2_S_3_ film. For the experimental group, the as‐prepared Precursor‐C films were further immersed in Solution B and deposited at 90°C for 15 min to construct a porous microstructure. The resulting Precursor‐PR films were finally crystallized using the identical annealing procedure to obtain the modified Sb_2_S_3_ film (denoted as Sb_2_S_3_‐PR). Meanwhile, the waste solution and precipitates generated during the CBD reaction were collected at different recycling cycles for subsequent utilization.

### 4.3 Device Fabrication

The as‐prepared Sb_2_S_3_‐C and Sb_2_S_3_‐PR films were further assembled into complete solar cells with a device configuration of FTO/CdS/Sb_2_S_3_/Spiro‐OMeTAD/Au. The Spiro‐OMeTAD hole‐transporting layer was deposited following the previous report [39]. Subsequently, the Au electrodes were thermally evaporated onto the Spiro‐OMeTAD layer, with an active area of 0.09 cm^2^.

### 4.4 Characterizations

The morphologies and the elemental ratio of the Sb_2_S_3_ films were performed by a double‐beam field emission scanning electron microscope (Dual Beam FEG SEM, Helios G4 CX) equipped with energy‐dispersive spectroscopy (EDS); The crystal structure of the Sb_2_S_3_ films were measured by X‐ray diffraction technology (XRD, Rigaku Smartlab −3 kW); A power meter (Keithley 2400) and a solar simulator (SUN 2000, ABET) were used to measure the current current–voltage (*J*–*V*) curves of the devices under a radiation density of 100 mW/cm^2^ and limited illumination area of 0.04 cm^2^; The external quantum efficiency (EQE) spectrum of the devices were tested by the QE system (Zolix SCS100, CROWNTECH) in range of 300–900 nm; The dark current‐voltage curves were performed by using a semiconductor tester (Fs‐Pro) with the DC bias voltage sweeps from −0.8 to 0.8 V; EIS measurements were performed using an electrochemical workstation (VPS) at a bias potential of 0.50 V in the dark with frequency ranging from 1 Hz to 1 MHz; Voltage‐dependent capacitance characteristic (C–V) and drive‐level‐capacitance‐profiling (DLCP) data were performed by using a semiconductor characterization system (Keithley 4200‐SCS, USA) at 50 kHz frequency and DC bias from –0.8 to 0.8 V; Ultraviolet photoelectron spectroscopy (UPS) of Sb_2_S_3_ films before and after PR strategy was performed with the ThermoFisher ESCALAB 250Xi spectrometer under a bias of –10 V; X‐ray Photoelectron Spectroscopy (XPS) was conducted on Thermofisher Scientific K‐Alpha with Al K_a_ radiation (energy: 1486.6 eV); The admittance spectra (AS) of Sb_2_S_3_ TFSCs were measured using a Keysight B1500A semiconductor characterization system equipped with a cryostat (PHY‐VPF‐100) over the temperature range of 100–350 K at 10 K intervals.

### 4.5 Calculation of Texture Coefficients for Crystal Planes in Sb_2_S_3_ Thin Film

To further concretize the vertical growth of Sb_2_S_3_ thin film under the function of PR strategy, the texture coefficient (*TC*) of Sb_2_S_3_‐C and Sb_2_S_3_‐PR films was calculated according to Equation (6) [40]:

(14) $$TC\left(hkl\right)=\frac{\frac{I_{\left(hkl\right)}}{I_{0\left(hkl\right)}}}{\left[\frac{1}{N}\left(\sum_{i=0}^{N}\frac{I_{\left(h_{i}k_{i}l_{i}\right)}}{I_{0\left(h_{i}k_{i}l_{i}\right)}}\right)\right]}$$

where *I*_(hkl)_ is the peak intensity of (*hkl*) plane in XRD pattern, *I*_0(hkl)_ is the standard peak intensity of the same plane in PDF card, *N* is the total number of peaks that taken into calculation.

### 4.6 Statistical Analysis

The statistical data of the Sb_2_S_3_ solar cells were analyzed using Origin 2024 software. The sample size for each statistical analysis was 25 (*n* = 25). Box plots for the device performance parameters (*V*_OC_, *J*_SC_, FF, PCE) were plotted, including the outlier screening, the calculation of the middle line, mean, interquartile range, and the normality checks. The distribution and variability of the data are depicted in the box plot. And the *V*_CPD_ of the device was analyzed using a normal distribution.

## Author Contributions

**Qiqiang Zhu**: software, Writing – review and editing, visualization. **Zixian Cai**: investigation, writing – original draft. **Ye Huang**: investigation. **Shuying Cheng**: resources, supervision, writing – review and editing. **Weihuang Wang**: conceptualization, writing – review and editing, methodology, formal analysis, validation, data curation, supervision, project administration, funding acquisition. **Jianhui Chen**: resources. **Qing Gao**: visualization, writing – review and editing, software. **Yeyang Lin**: investigation, writing – original draft. **Mingen Zheng**: investigation, writing – original draft.

## Conflicts of Interest

The authors declare no conflicts of interest.

## Supporting information




**Supporting File**: advs76438‐sup‐0001‐SuppMat.docx.
