# Supplementary material for: High‐Efficiency Precipitate Recycling Strategy Paves the Way for Efficient Sb2S3 Solar Cells Fabricated by CBD Method
Source: Adv Sci (Weinh). 2026 Jul 11:e76438. Online ahead of print. doi: 10.1002/advs.76438 (PMC13355936; doi:10.1002/advs.76438)
Supplement: Supplementary file 1 — Supporting File: advs76438‐sup‐0001‐SuppMat.docx. [file ADVS-9999-e76438-s001.docx]

**Supporting Information**

**High-Efficiency Precipitate Recycling Strategy Paves the Way for Efficient Sb_2_S_3_ Solar Cells Fabricated by CBD Method**

Yeyang Lin^1^, Zixian Cai^1^, Mingen Zheng^1^, Ye Huang^1^, Weihuang Wang^1,3,^*, Qiqiang Zhu^1,^*, Qing Gao^2,*^, Jianhui Chen^2^, Shuying Cheng^1,3,*^

^1^Institute of Micronano Devices & Solar Cells, College of Physics & Information Engineering, Fuzhou University, Fuzhou 350108, Fujian, China

^2^Hebei Key Lab of Optic-Electronic Information and Materials, Department of Physics Science and Technology, Hebei University, Baoding 071002, Hebei, China

^3^Jiangsu Collaborative Innovation Center for Photovoltaic Science and Engineering, Changzhou 213164, Peoples Republic of China

***Corresponding authors**

E-mail addresses: [weihuangwang@fzu.edu.cn](mailto:weihuangwang@fzu.edu.cn) (W. Wang); qiqiangzhu@foxmail.com (Q. Zhu); [gaoqing@hbu.edu.cn (Q](mailto:gaoqing@hbu.edu.cn%20(Q). Gao); [sycheng@fzu.edu.cn](mailto:sycheng@fzu.edu.cn;yizhang@nku.edu.cn) (S. Cheng)


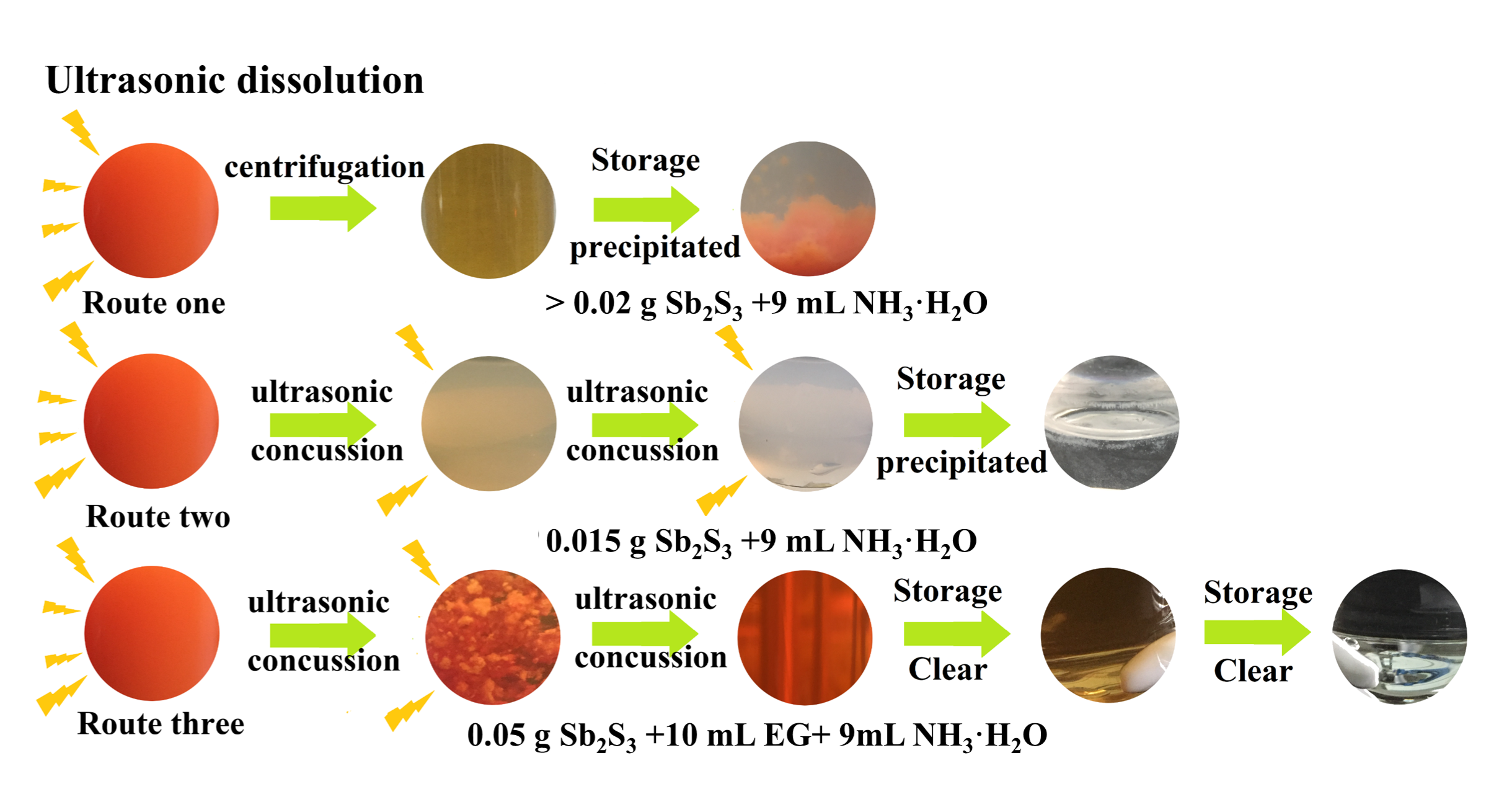


**Figure S1.** Sb_2_S_3_ dissolution processes under different conditions.


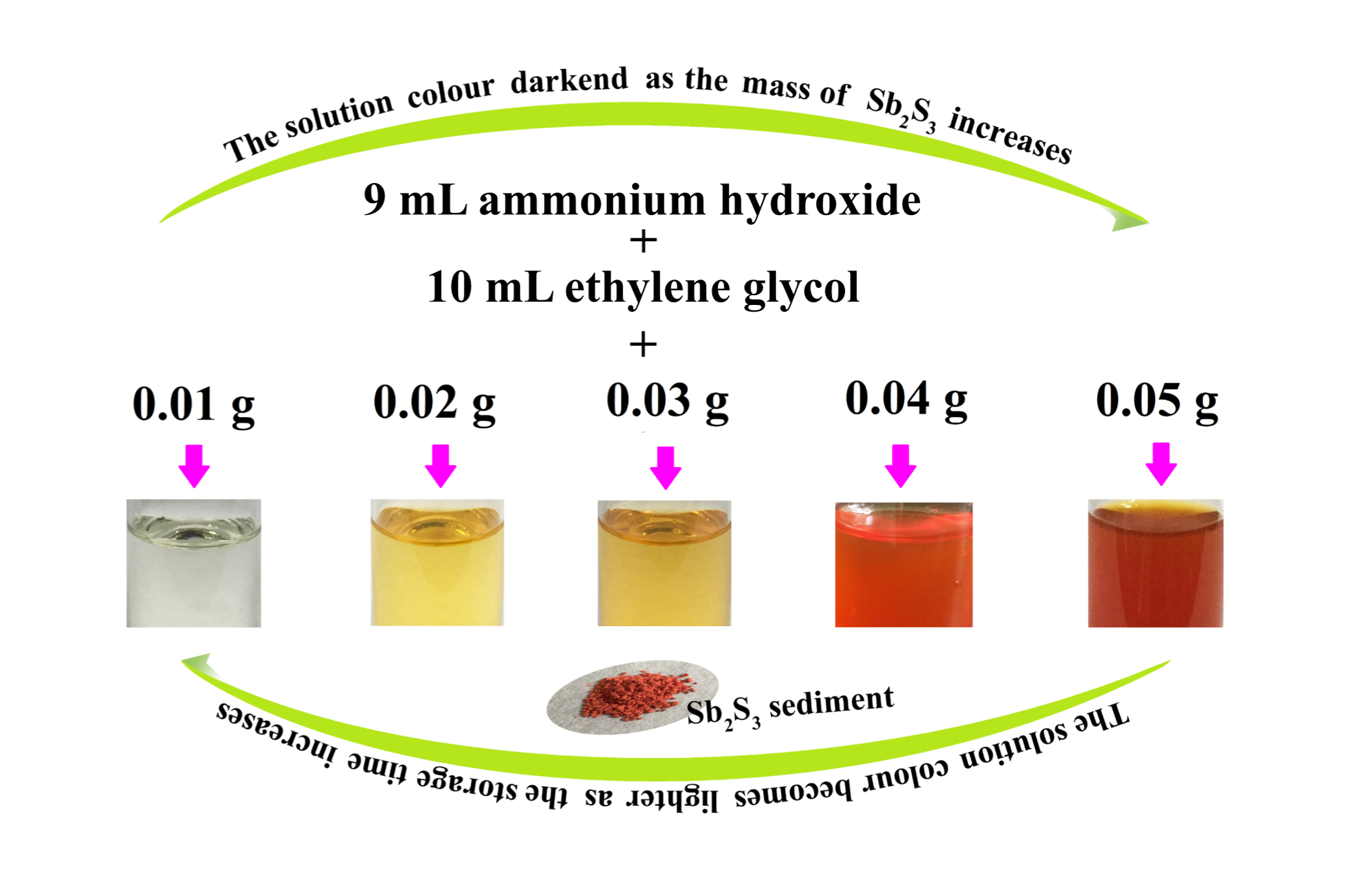


**Figure S2.** Dissolution features of Sb_2_S_3_ precipitates in ammonia/ethylene glycol mixtures.


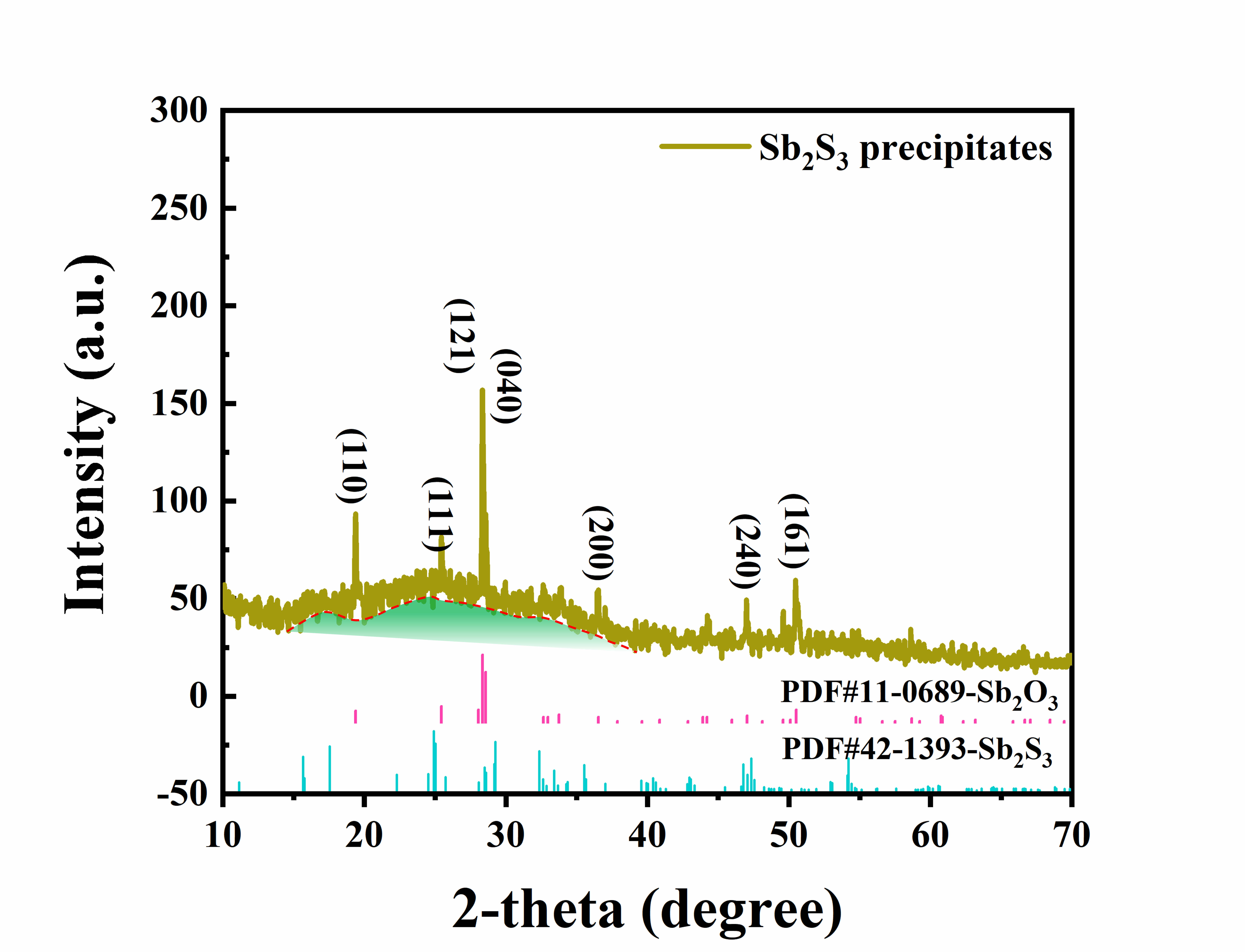


**Figure S3.** XRD pattern of the collected first-cycle Sb_2_S_3_ precipitates.


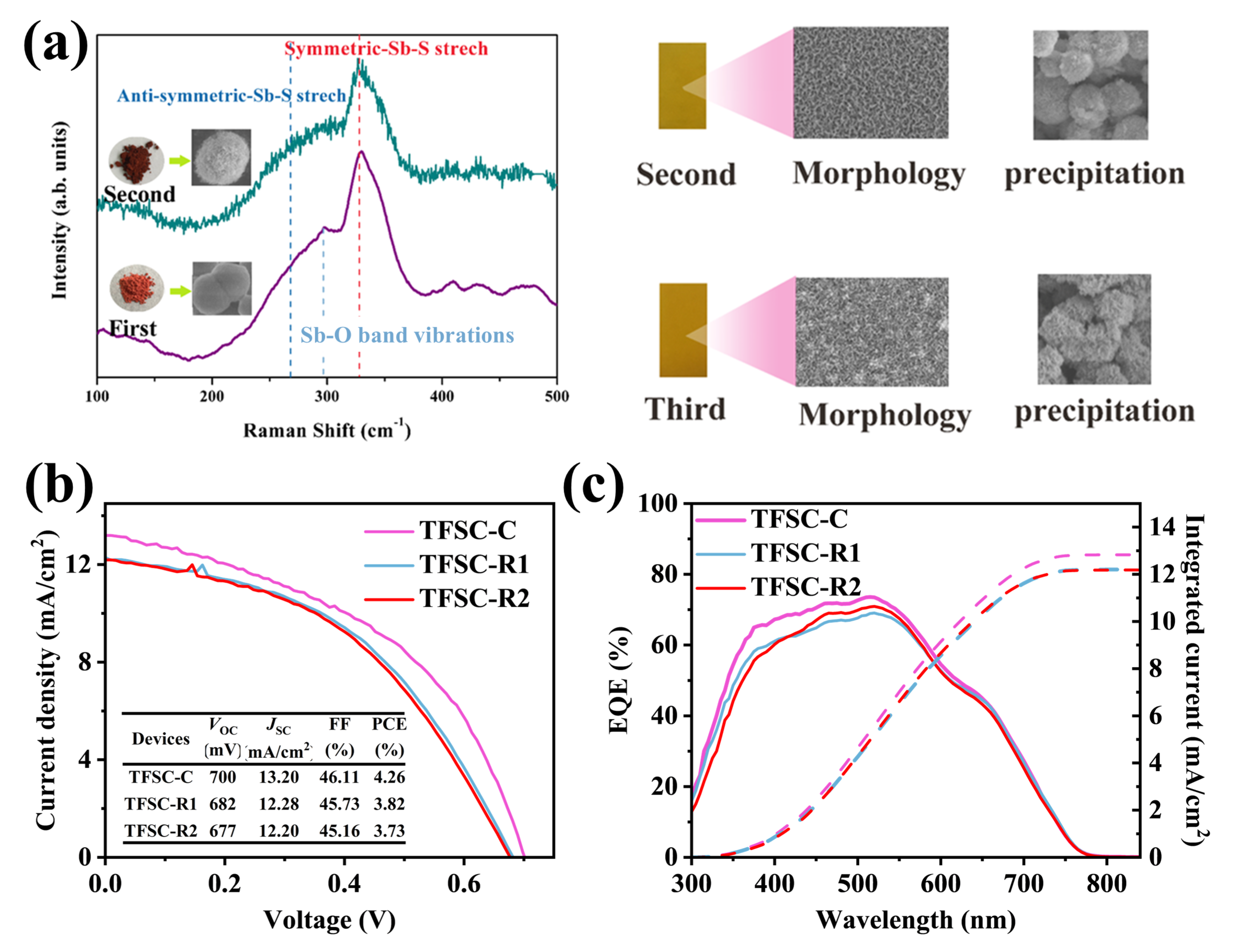


**Figure S4.** (a) Raman spectra and SEM morphologies of the Sb_2_S_3_precipitates produced during different deposition cycle; (b) Light *J-V* and (c) EQE curves of TFSC-C, TFSC-R1 and TFSC-R2 devices.


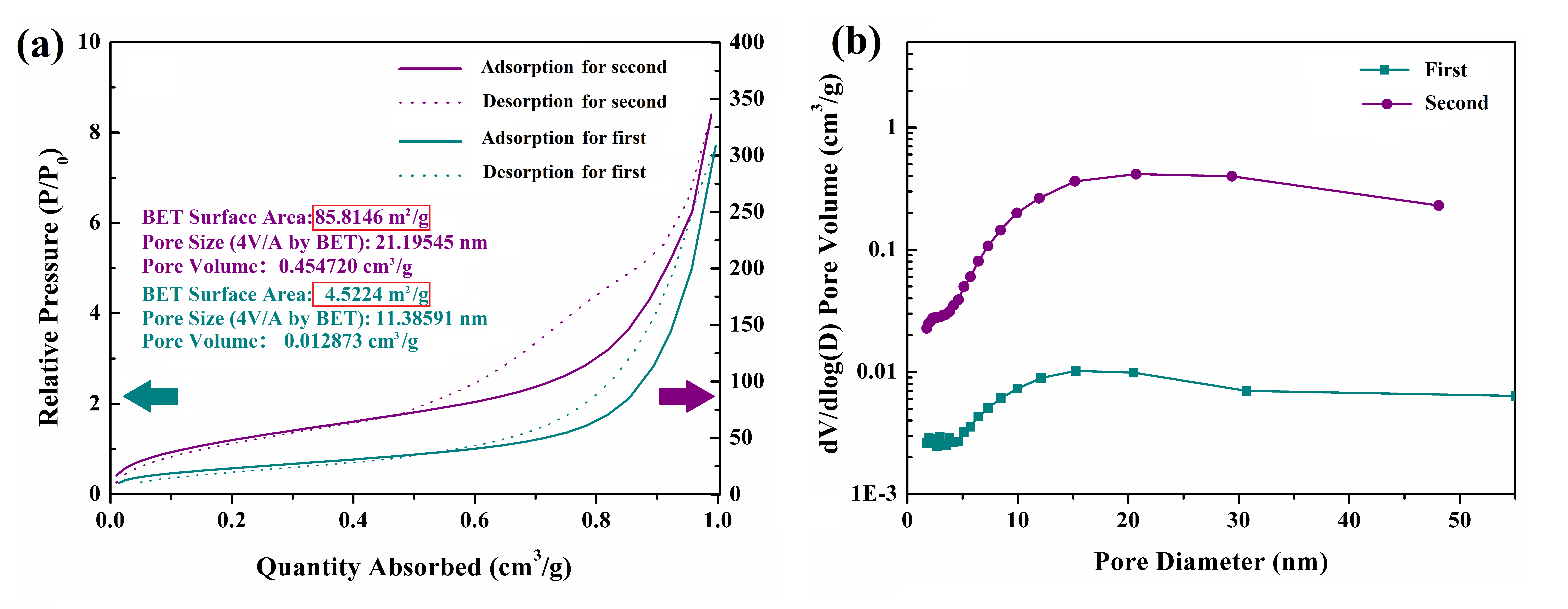


**Figure S5.** (a) N_2_ adsorption-desorption isotherms and (b) pore size distribution of Sb_2_S_3_precipitates produced during the first and second deposition process.


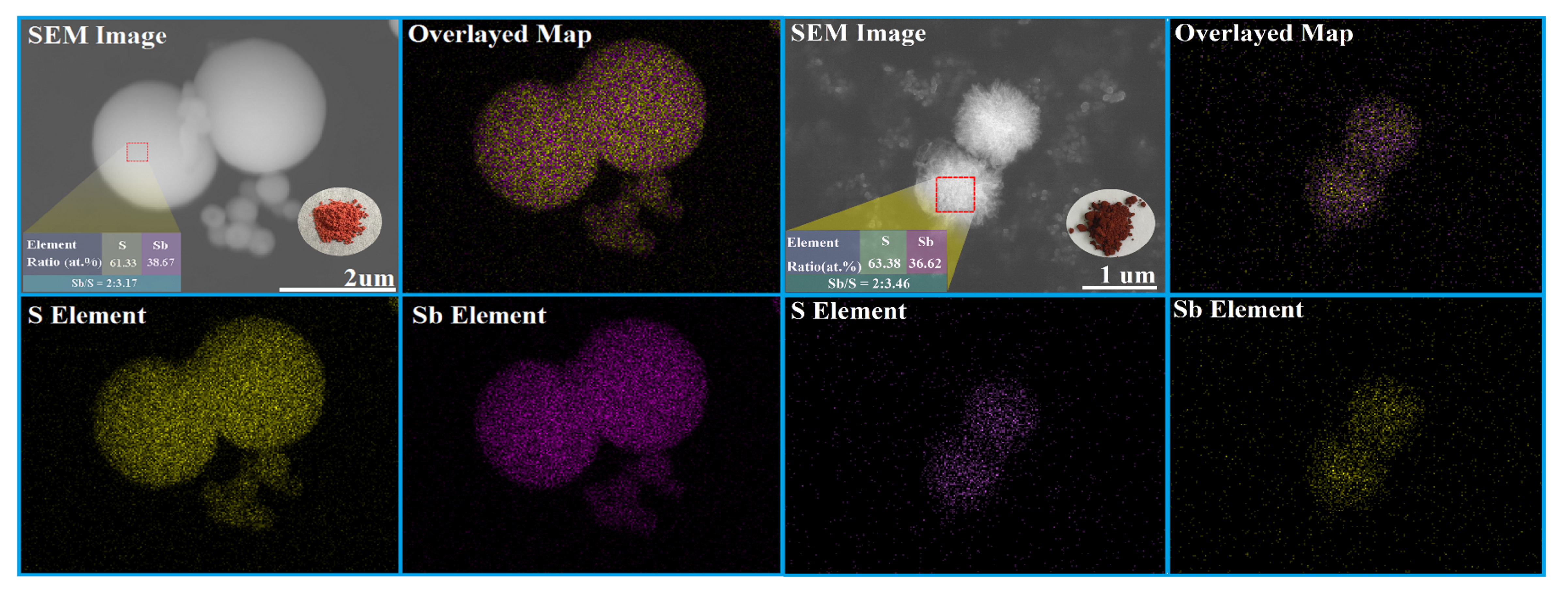


**Figure S6.** EDS elemental mappings of the Sb_2_S_3_precipitates produced during different deposition cycle.


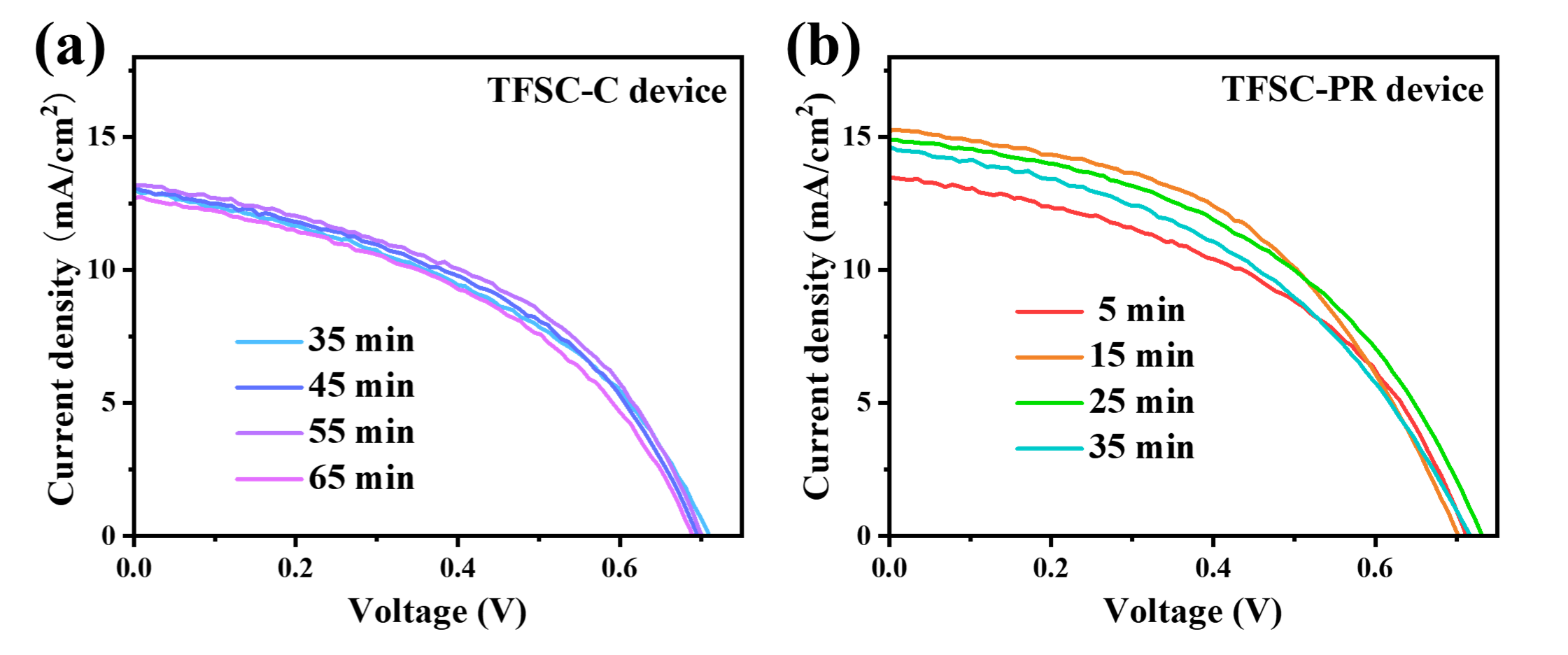


**Figure S7.** (a) *J*-*V* curves of TFSC-C devices with different deposition time; (b) *J*-*V* curves of TFSC-PR devices with different secondary deposition time.

**Table S1**. Performances of TFSC-C devices with different deposition time.

| **Deposition time**  **(min)** | ***V*_OC_**  **(mV)** | ***J*_SC_**  **(mA/cm^2^)** | **FF**  **(%)** | **PCE**  **(%)** |
| --- | --- | --- | --- | --- |
| **35 min** | 711 | 13.01 | 42.94 | 3.97 |
| **45 min** | 696 | 13.11 | 44.66 | 4.08 |
| **55 min** | 700 | 13.20 | 46.11 | 4.26 |
| **65 min** | 690 | 12.72 | 43.92 | 3.86 |

**Table S2** Performances of TFSC-PRM devices with different secondary deposition time.

| **Deposition duration**  **(min)** | ***V*_OC_**  **(mV)** | ***J*_SC_**  **(mA/cm^2^)** | **FF**  **(%)** | **PCE**  **(%)** |
| --- | --- | --- | --- | --- |
| **5 min** | 713 | 13.49 | 46.11 | 4.43 |
| **15 min** | 702 | 15.25 | 48.15 | 5.15 |
| **25 min** | 732 | 14.90 | 45.99 | 5.02 |
| **35 min** | 718 | 14.62 | 43.59 | 4.57 |


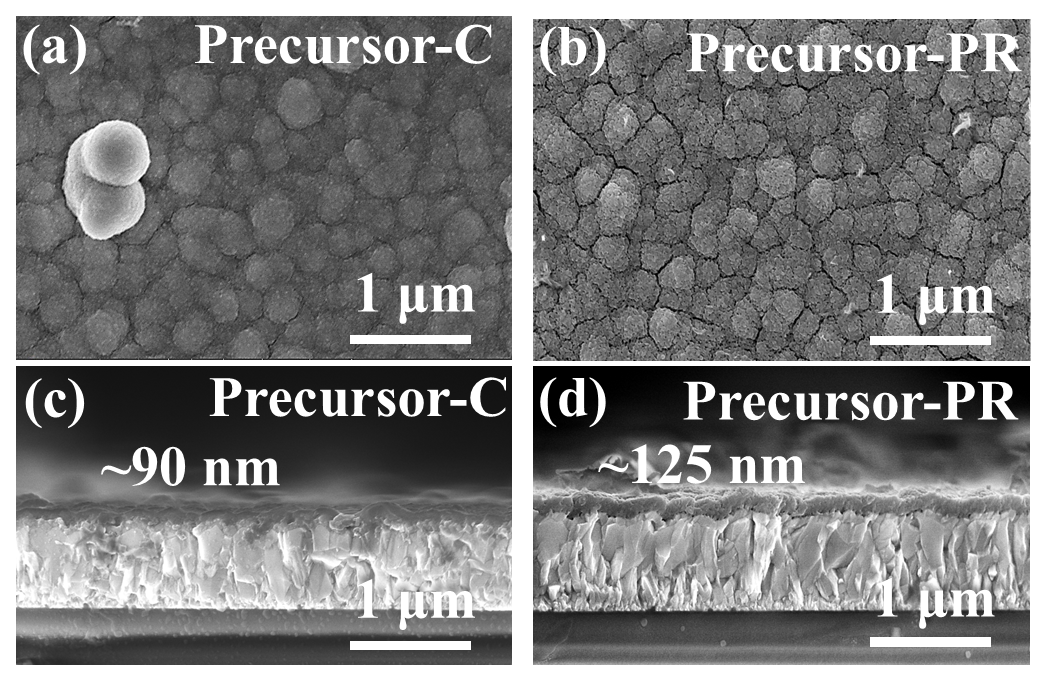


**Figure S8.** SEM images of the as-deposited Precursor-C and Precursor-PR films.





**Figure S9.** The grazing incidence X-ray diffraction (GIXRD) patterns of Sb_2_S_3_-C and Sb_2_S_3_-PR films.


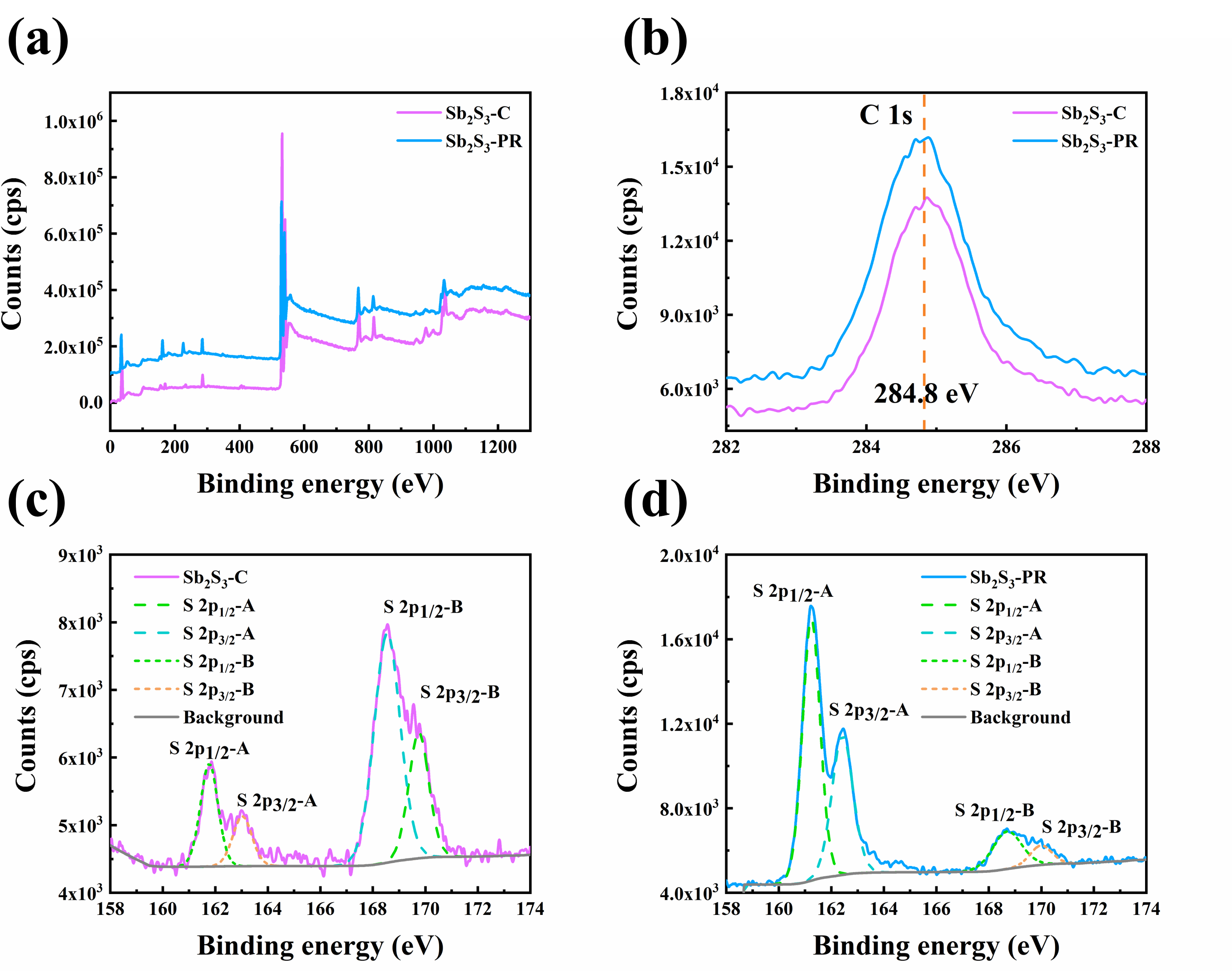


**Figure S10.** XPS characteristics of the Sb_2_S_3_ films: (a) survey spectra, high-resolution core-level scans of (b) C 1s for Sb_2_S_3_ films, and the S 2p for the (c) Sb_2_S_3_-C and (d) Sb_2_S_3_-PR films.


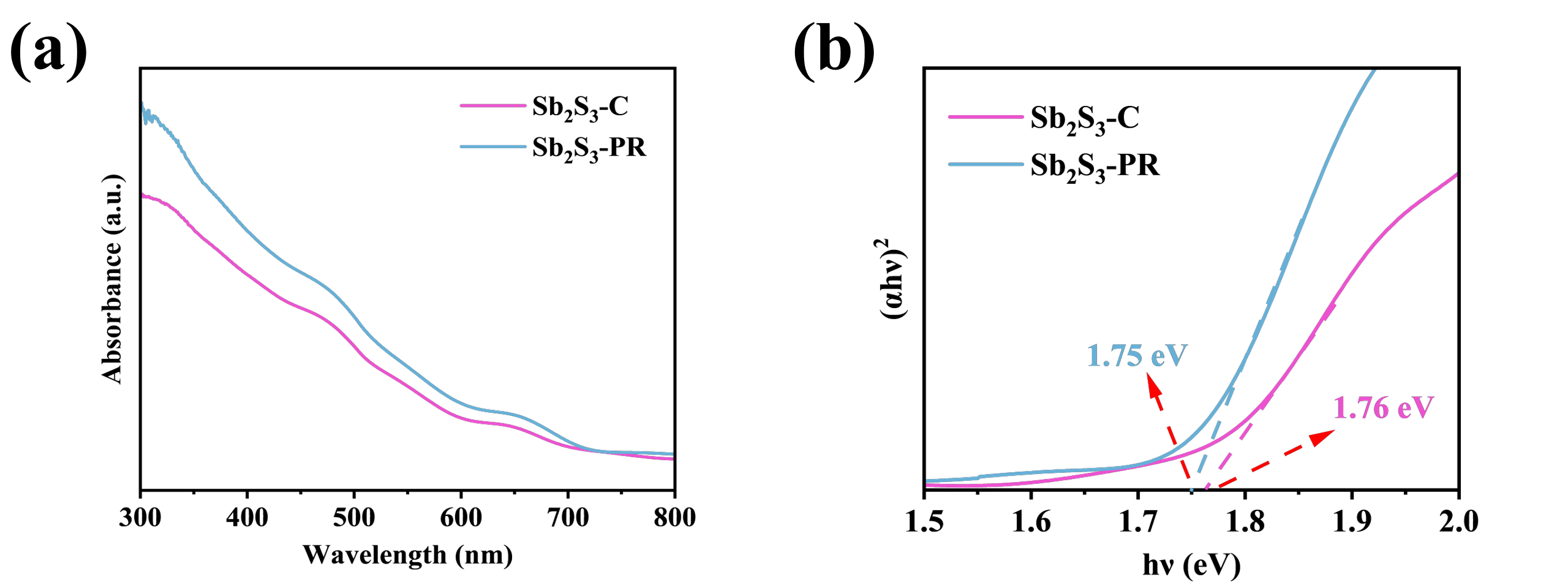


**Figure S11.** (a) UV-Vis absorption spectra and (b) corresponding Tauc plots of Sb_2_S_3_-C and Sb_2_S_3_-PR films.


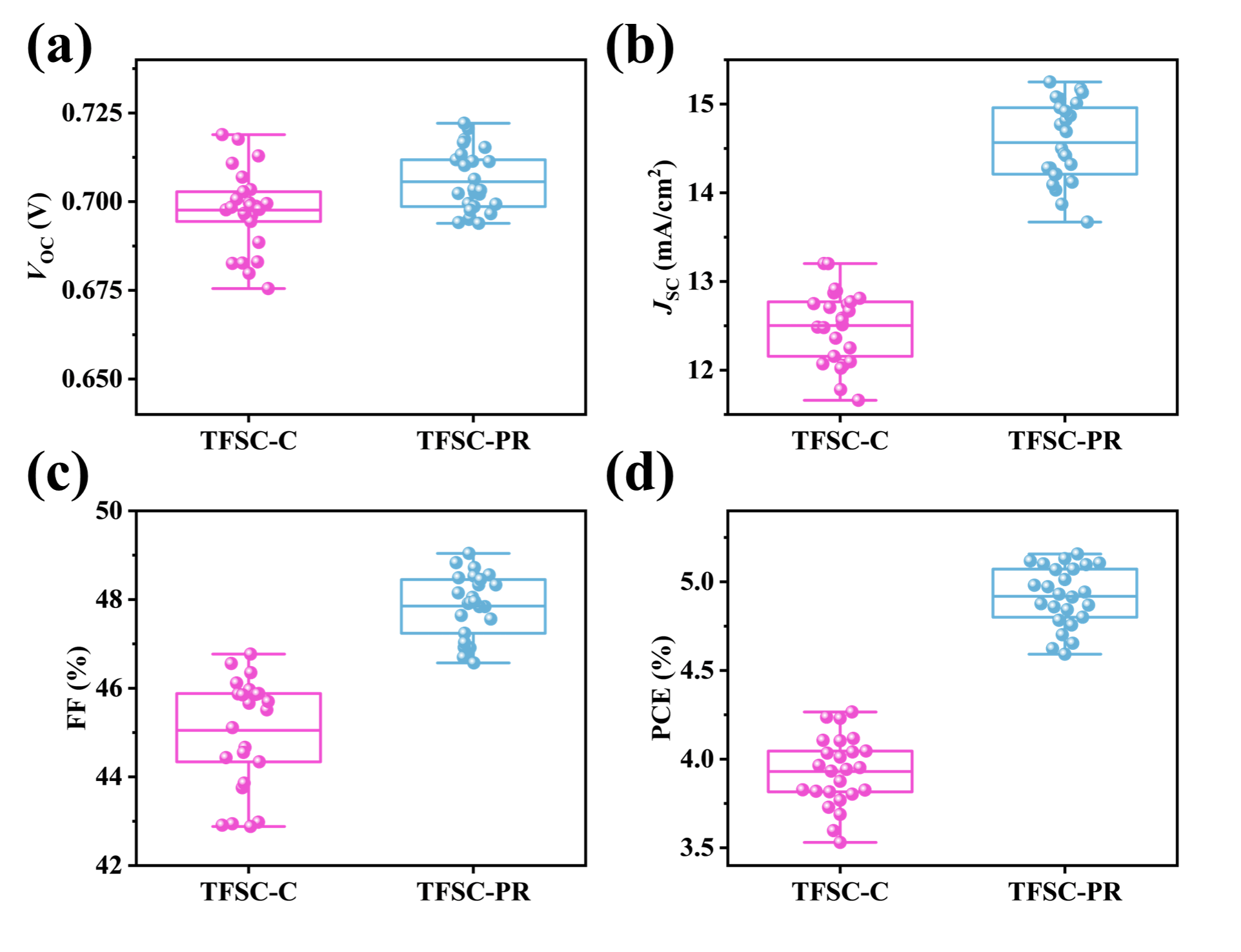


**Figure S12.** Statistical parameters of TFSC-C and TFSC-PR devices: (a) *V*_OC_, (b) *J*_SC_, (c) FF, and (d) PCE.

**Table S3** Statistical results of the device parameters for TFSC-C and TFSC-PR devices.

| **Device** | ***V*_OC_**  **(mV)** | ***J*_SC_**  **(mA/cm^2^)** | **FF**  **(%)** | **PCE**  **(%)** |
| --- | --- | --- | --- | --- |
| **TFSC-C** | 698 ± 11 | 12.50 ± 0.41 | 45.05 ± 1.23 | 3.93 ± 0.19 |
| **TFSC-PR** | 706 ± 9 | 14.57 ± 0.45 | 47.85 ± 0.72 | 4.92 ± 0.17 |


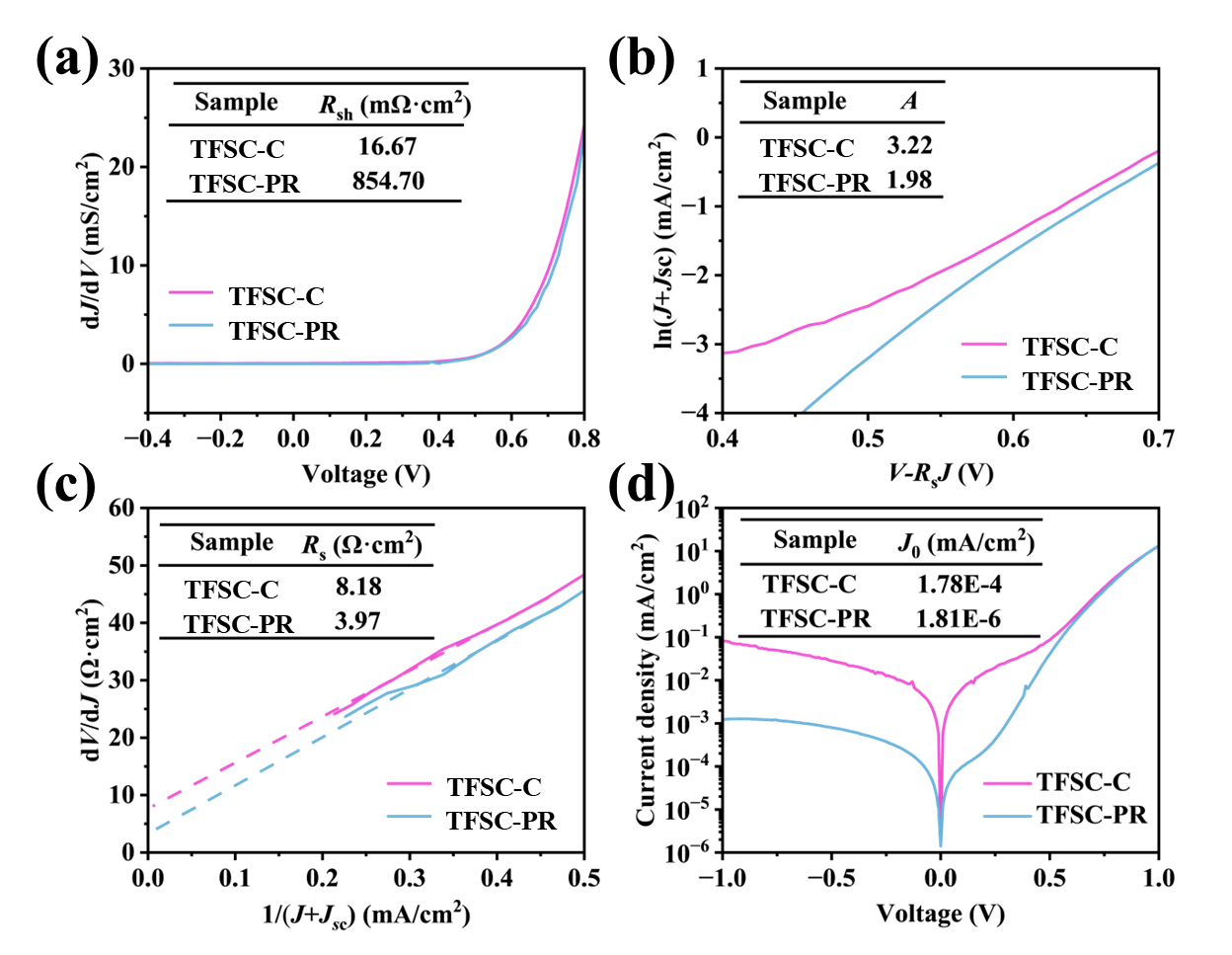


**Figure S13.** Dark *J*–*V* characteristics of the Sb_2_S_3_ devices: (a) shunt characterization *G*(V); (b) ln(*J*+*J*_SC_-*GV*) against *V*-*R*_S_*J*; (c) d*V*/d*J* against (*J*+*J*_SC_-*GV*)^-1^; (d) dark *J-V* curves.


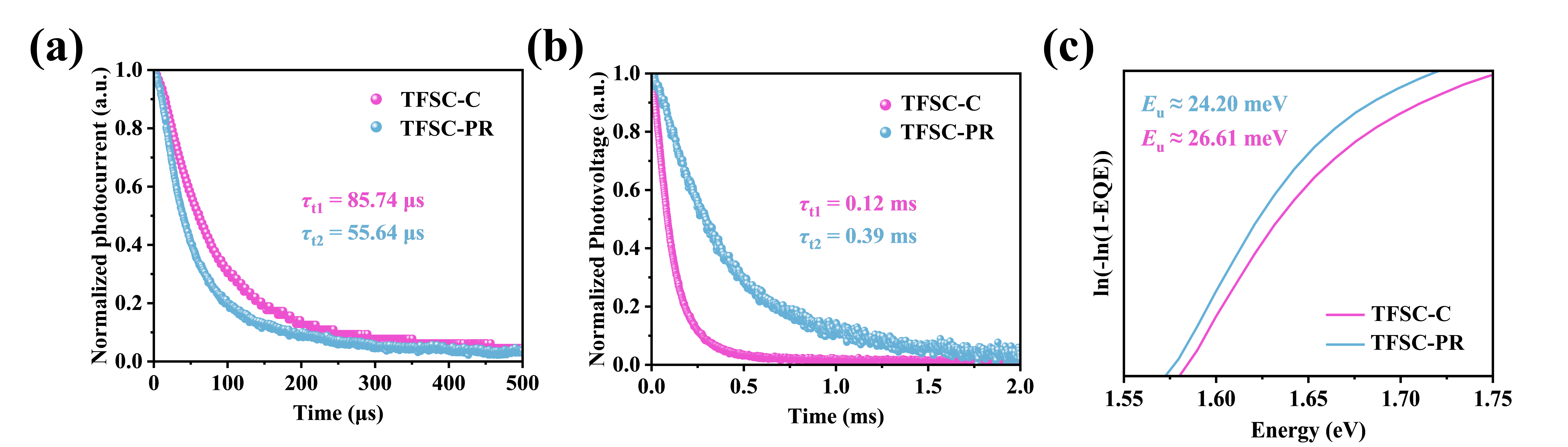


**Figure S14.** (a) Transient photocurrent plots, (b) transient photovoltage plots, and (c) Urbach energy plots of TFSC-C and TFSC-PR devices.


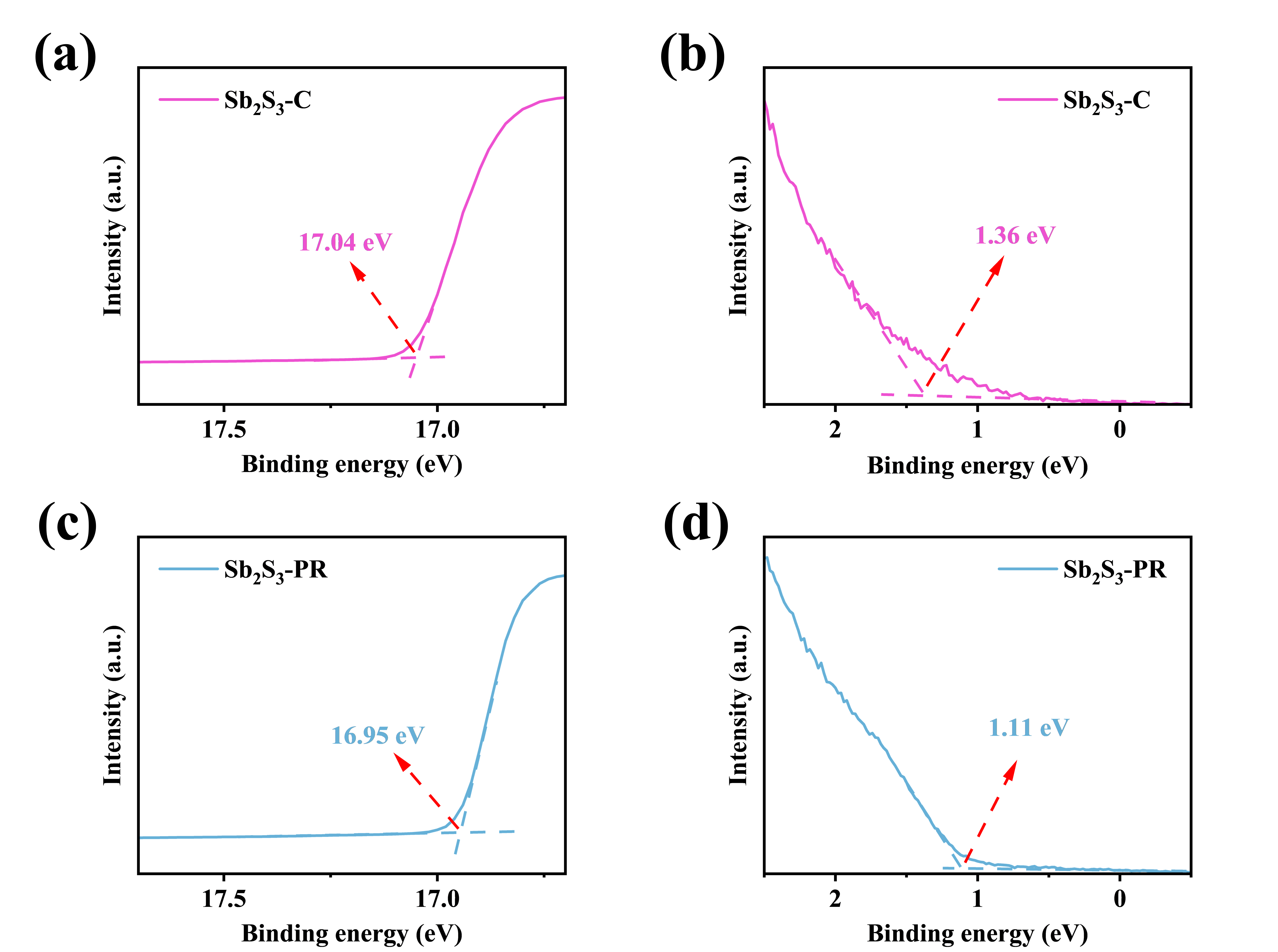


**Figure S15.** UPS onset and cutoff spectra of (a/b) Sb_2_S_3_-C film and (c/d) Sb_2_S_3_-PR film.


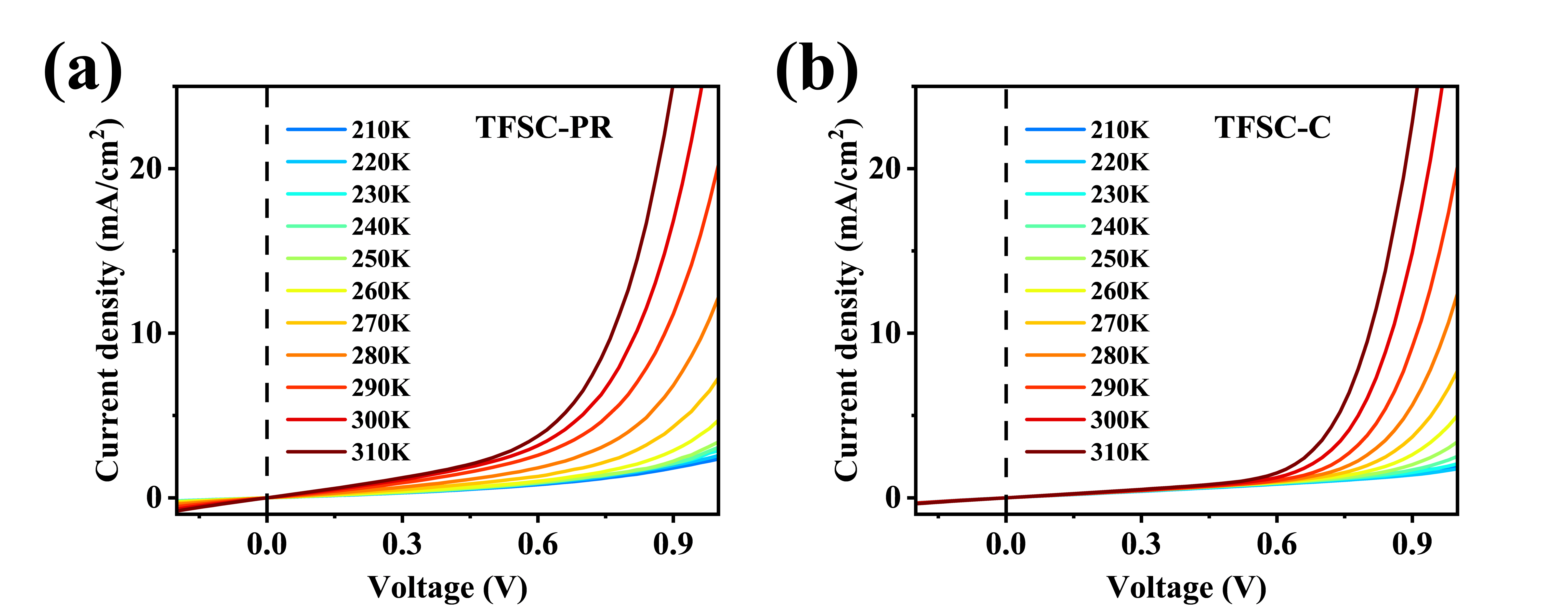


**Figure S16.** Temperature dependent dark *J-V-T* curves of (a) TFSC-PR and (b) TFSC-C devices.
